# Critical role of sphingosine-1-phosphate receptor-2 in the disruption of cerebrovascular integrity in experimental stroke

**DOI:** 10.1038/ncomms8893

**Published:** 2015-08-05

**Authors:** Gab Seok Kim, Li Yang, Guoqi Zhang, Honggang Zhao, Magdy Selim, Louise D. McCullough, Michael J. Kluk, Teresa Sanchez

**Affiliations:** 1 Department of Emergency Medicine, the Center for Vascular Biology Research, Beth Israel Deaconess Medical Center, Harvard Medical School, Boston, Massachusetts 02215, USA; 2 Department of Surgery, the Center for Vascular Biology Research, Beth Israel Deaconess Medical Center, Harvard Medical School, Boston, Massachusetts 02215, USA; 3 Department of Neurology, Beth Israel Deaconess Medical Center, Harvard Medical School, 330 Brookline Avenue, RN-227A, Boston, Massachusetts 02215, USA; 4 Department of Neurology, University of Connecticut School of Medicine, Farmington, Connecticut 06030, USA; 5 Department of Pathology, Brigham and Women’s Hospital, Harvard Medical School, Boston, Massachusetts 02215, USA

## Abstract

The use and effectiveness of current stroke reperfusion therapies are limited by the complications of reperfusion injury, which include increased cerebrovascular permeability and haemorrhagic transformation. Sphingosine-1-phosphate (S1P) is emerging as a potent modulator of vascular integrity via its receptors (S1PR). By using genetic approaches and a S1PR2 antagonist (JTE013), here we show that S1PR2 plays a critical role in the induction of cerebrovascular permeability, development of intracerebral haemorrhage and neurovascular injury in experimental stroke. In addition, inhibition of S1PR2 results in decreased matrix metalloproteinase (MMP)-9 activity *in vivo* and lower gelatinase activity in cerebral microvessels. S1PR2 immunopositivity is detected only in the ischemic microvessels of wild-type mice and in the cerebrovascular endothelium of human brain autopsy samples. *In vitro*, S1PR2 potently regulates the responses of the brain endothelium to ischaemic and inflammatory injury. Therapeutic targeting of this novel pathway could have important translational relevance to stroke patients.

## Abstract (web summary)

The sphingolipid sphingosine-1-phosphate (S1P) plays a vital role in vascular homeostasis through the interaction with its receptors S1PR1-5. Here, by using genetic and pharmacological approaches, the authors show that S1PR2-mediated signaling is crucial for the disruption of cerebrovascular integrity and development of intracerebral haemorrhage in a mouse stroke model.

## Graphical abstract

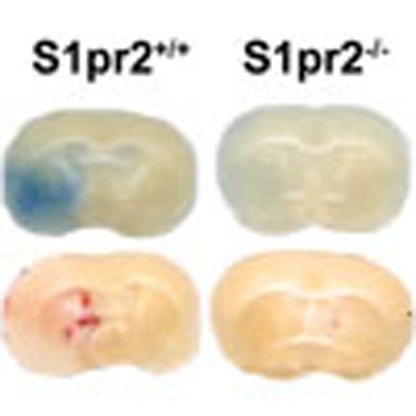

Stroke is a leading cause of death and long-term disability worldwide^1^. Despite many decades of intensive research, therapeutic options for patients presenting with ischaemic stroke are very limited. Neuroprotective agents have been designed to block one or more steps of the neuronal ischaemic cascade. However, none of these drugs unequivocally has shown an improvement in clinical outcomes^2^,^3^. Currently, stroke therapies aim at establishing early reperfusion by thrombolytic (for example, tissue plasminogen activator) and/ or mechanical recanalization of obstructed blood vessel/s^4^,^5^. However, restoration of blood flow exacerbates brain injury due to increased reactive oxygen species generation and further activation of matrix metalloproteinases (MMP) with subsequent development of vasogenic oedema (increased cerebrovascular permeability) and haemorrhagic transformation^5^,^6^, two life-threatening complications, which limit the use and effectiveness of stroke reperfusion strategies. Administration of vasoprotective agents at the time of reperfusion could potentially decrease the risk and the extent of these complications by improving the integrity of the injured cerebral blood vessels, which could ultimately result in improved stroke outcomes.

Ischaemia–reperfusion (I/R) injury induces metabolic stress and inflammatory responses in the neurovascular unit. Although neurons are extremely sensitive to the lack of oxygen and nutrients, I/R injury also compromises the barrier properties of brain endothelial cells, which contributes to neurovascular pathophysiology by allowing the entrance of toxic plasma proteins and blood cells into the brain parenchyma^7^, increasing intracranial pressure and compromising the delivery of nutrients and oxygen. In the acute phase of stroke (minutes to hours), reactive oxygen species and pro-inflammatory cytokines released in the brain parenchyma, such as tumour necrosis factor-α (TNF-α) or interleukin-1β, are known to induce vascular permeability, adhesion molecule expression, and the expression and activity of MMP, which exacerbate blood–brain barrier disruption, worsening neurovascular injury^8^,^9^. In the subacute phase, further disruption of blood vessel integrity occurs, leading to haemorrhagic transformation and exacerbation of neuronal injury^5^. In experimental models of stroke, activation of MMP and the subsequent degradation of endothelial basal lamina and tight junction proteins have been shown to play a critical role in the disruption of cerebrovascular integrity^8^,^10^,^11^. Since MMPs can be highly toxic molecules, they are tightly regulated at the transcriptional, translational and post-translational levels. Among MMP, MMP-2 is constitutively expressed in the brain and brain endothelial cells, while basal MMP-9 levels are very low and rapidly induced by pro-inflammatory stimuli^12^. Although it is well-established that MMP contribute to neurovascular injury in stroke, currently, there are no therapies specifically targeting MMP, due to the complex regulation of MMP activity, as well as the lack of specificity of MMP inhibitors and their numerous side effects.

The bioactive sphingolipid, sphingosine-1-phosphate (S1P) is emerging as a potent modulator of vascular integrity. S1P is present at high levels (within the range of several hundred nanomolars) in plasma and lymph. It is generated from the metabolism of sphingomyelin through the actions of sphingomyelinase, ceramidase and sphingosine kinase (SPHK). Although platelets store high amounts of S1P, the main sources of plasma S1P are erythrocytes^13^ and endothelial cells^14^, which exhibit high SPHK activity. S1P regulates many cellular functions via its interaction with the endothelial differentiation gene family of G protein-coupled receptors, renamed as S1PR1-5 (ref. 15). S1P binds to its receptors within the low nanomolar range (the *K*_D_ of S1P for S1PRs ranges from 8 to 27 nM). S1PR activate different intracellular signalling pathways and differentially regulate endothelial cell function. S1PR1 couples to G_i_ and activates the phosphatidylinositol-3-kinase (PI3K) pathway^16^, which promotes vascular integrity^17^,^18^,^19^. In contrast, we found that S1PR2 antagonizes S1PR1–G_i_–PI3K signalling in the endothelium through activation of the G_12_–Rho pathway^20^ and that S1PR2 is sufficient for endothelial activation *in vitro*^21^. The role of S1P in vascular development is well-illustrated by S1pr1 null mice, which exhibit a defect in vascular maturation^18^. In adult mice, S1PR1 promotes vascular integrity^19^ and regulates lymphocyte trafficking^22^. In fact, the recently FDA-approved Fingolimod, is a potent immunosuppressant via S1PR1. In contrast to S1PR1, S1PR2 is not required for embryonic vascular development and *S1pr2*^*−/−*^ mice are viable and develop normally^23^. In addition, using a model of acute inflammatory vascular injury, we recently reported that S1PR2 expression in the stromal/endothelial compartment, and not in haematopoietic cells, is critical for the induction of vascular permeability, endothelial activation and sustained systemic inflammation^21^, suggesting that S1PR2 could be a novel target for acute vascular protection in various clinical settings.

Herein, we aimed to assess the role of S1PR2 in the regulation of cerebrovascular integrity after I/R injury. Using a mouse model of focal transient cerebral ischaemia, the middle cerebral artery occlusion model (MCAO), we report the critical role of S1PR2 in the disruption of cerebrovascular integrity after I/R injury. S1PR2 is induced in cerebral microvessels early after I/R injury. Our *in vitro* studies with mouse and human primary brain microvascular endothelial cells (hBMVEC), mouse primary neurons and mouse primary mixed glial cells provide novel mechanistic insights into the regulation of cerebrovascular responses to I/R injury by S1PR2. Finally, we detect S1PR2 in the cerebrovascular endothelium in human autopsy specimens. Altogether our data point to S1PR2 as a novel therapeutic target for acute cerebrovascular protection in stroke.

## Results

### Critical role of S1PR2 in neuronal injury in stroke

**Figure 1:**
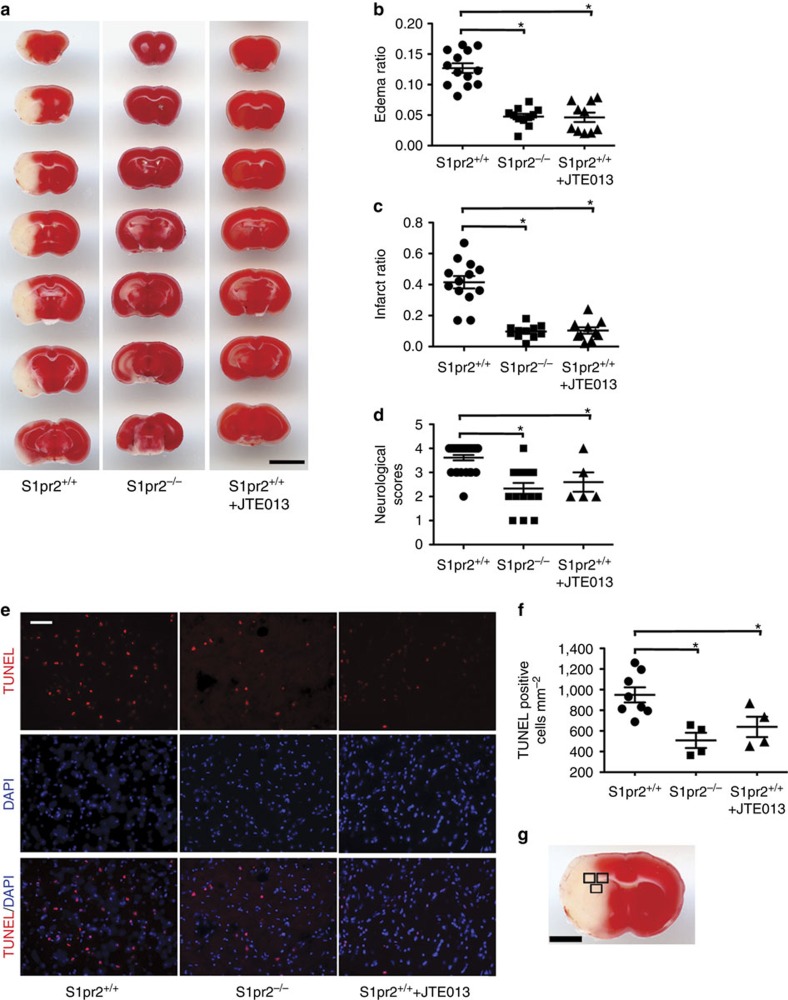
Inhibition of S1PR2 results in less oedema and neuronal injury in experimental stroke. Ischaemia (90 min.) was induced in wild-type (*S1pr2*^*+/+*^) and *S1pr2*^*−/−*^ by MCAO. After reperfusion, mice received vehicle or the S1PR2 antagonist, JTE013 (30 mg kg^−1^), by gavage. (**a**) Representative images of TTC staining of seven, 1-mm-thick brain coronal slices 24 h after reperfusion. Scale bar, 5 mm. (**b**) Oedema and (**c**) infarct ratios were calculated by image analysis and reported as a ratio of the non-ischaemic hemisphere. Infarct ratios were corrected for oedema. The individual values and the mean±s.e.m. are shown. *N*=10–13 from eight independent experiments. *P<0.05 (one-way ANOVA followed by Newman–Keuls). (**d**) Improved neurological scores (that is, lower neurological deficit scores) were observed in *S1pr2*^*−/−*^ or JTE013-treated *S1pr2*^*+/+*^ mice compared with vehicle-treated *S1pr2*^*+/+*^, 24 h after reperfusion. (**e**) Representative pictures of TUNEL assay (red channel) in the damaged area from *S1pr2*^*+/+*^, *S1pr2*^*−/−*^ and JTE013-treated *S1pr2*^*+/+*^ mice. Blue channel: nuclear staining (DAPI). (**f**) Quantification of TUNEL-positive cells per mm^2^. Values are the average of TUNEL-positive cells from three fields. *n*=4–8 from four independent experiments. Scale bar, 50 μm. (**g**) Image showing the anatomical areas in which TUNEL-positive cells were quantified in mice (bregma +1.2 to +0.8 mm, rostrally). Scale bar, 2.5 mm.

To study the role of S1PR2 signalling in the regulation of neurovascular responses to I/R injury, we used a mouse model of transient focal cerebral ischaemia, the transient MCAO (tMCAO) mouse model of stroke. *S1pr2*^*+/+*^ and *S1pr2*^*−/−*^ littermates were subjected to transient focal ischaemia for 90 min. After 24 h of reperfusion, infarct and oedema ratios were quantified in wild-type and *S1pr2*^*−/−*^ mice as previously described^24^. As shown in Fig. 1a–c, *S1pr2*^*−/−*^ mice exhibited a dramatic decrease in both infarct ratio (wild-type 0.324± 0.053 versus S1pr2 null 0.084±0.017, 74% reduction, *P*<0.05, one-way analysis of variance (ANOVA) followed by Newman–Keuls test) and total cerebral oedema ratio (wild-type 0.121± 0.011 versus *S1pr2*^*−/−*^ 0.047±0.008, 61.2% reduction, *P*<0.05, one-way ANOVA followed by Newman–Keuls test), which is the sum of cytotoxic and vasogenic oedema. In addition, *S1pr2*^*−/−*^ mice exhibited improved neurological scores compared with wild-type (Fig. 1d). We also found that the infarct size was significantly reduced and the neurological scores were significantly improved in *S1pr2* null mice compared with wild-type, 5 days after tMCAO (Supplementary Fig. 1a–d).

To evaluate the therapeutic targeting of S1PR2 in stroke reperfusion therapies, we acutely inhibited S1PR2 activation by administration of the S1PR2 antagonist, JTE013 by gavage. In agreement with our data in *S1pr2* null mice, administration of the S1PR2 antagonist (30 mg kg^−1^) 10 min after reperfusion (1.7 h after the onset of ischaemia) in wild-type mice resulted in a dramatic decrease in both infarct and oedema ratios (79.6% and 81.03% inhibition, respectively, Fig. 1a–c), as well as in a significant improvement of the neurological scores (Fig. 1d). Consistent with our recent studies using sepsis models^21^, the minimum effective dose of JTE013 was 30 mg kg^−1^ (Supplementary Fig. 2). Mortality was <5% in all cohorts of mice 24 h after reperfusion.

Terminal deoxynucleotidyl transferase dUTP nick end labelling (TUNEL) assays showed a significant decrease in TUNEL-positive cells both in *S1pr2*^*−/−*^ and JTE013-treated *S1pr2*^*+/+*^mice (508.7±74 and 639±98 positive cells per mm^2^, respectively) compared with vehicle-treated wild-type mice (949±73 positive cells per mm^2^) (Fig. 1e,f), indicating that inhibition of S1PR2 decreases DNA fragmentation that results from apoptosis after I/R injury. No TUNEL-positive cells were detected in the contralateral hemisphere (not shown).

**Figure 2:**
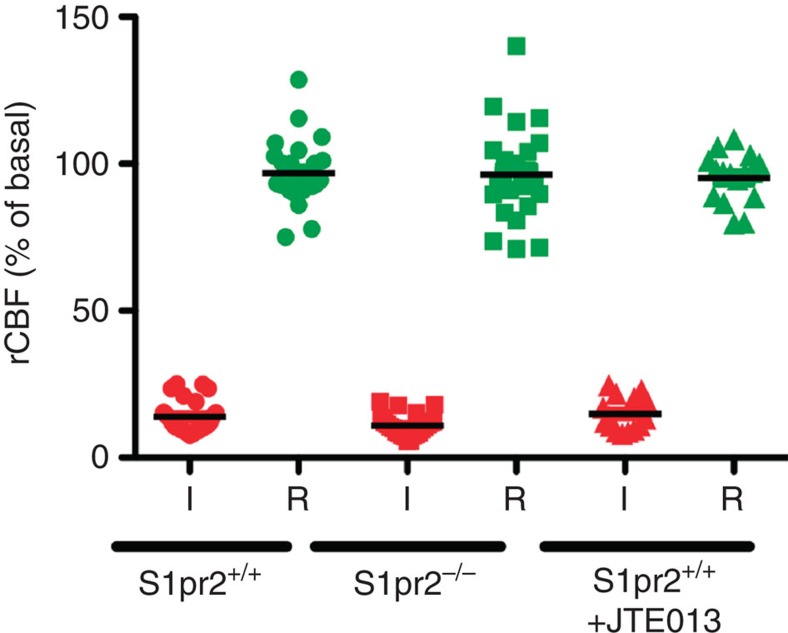
Regional cerebral blood flow (CBF) in *S1pr2*^*+/+*^ and *S1pr2*^*−/−*^ mice. Regional CBF in the middle cerebral artery territory was measured throughout the surgeries using a laser Doppler probe, attached to the temporal bone. The relative CBF (% of basal) during MCA occlusion (I, red circles, squares and triangles) and 10 min after reperfusion (R, green circles, squares and triangles) is shown in *S1pr2*^*+/+*^-, *S1pr2*^*−/−*^- and *S1pr2*^*+/+*^-receiving JTE013 is shown. *N*=18–30.

No significant differences were observed in the physiological parameters (arterial O_2_ saturation, heart rate, pulse distention and respiratory rate) between wild-type and *S1pr2*^*−/−*^ mice, before, during or after MCAO (Table 1). In addition, cerebral blood flow (CBF) in the territory of the MCA, monitored during the surgeries by Laser Doppler, was similarly reduced in all three groups of mice during occlusion (13%±1.2 of basal level) and similarly restored after reperfusion in wild-type, *S1pr*^*−/−*^ and JTE013-treated wild-type mice (94.6%±1.1 of basal) (Fig. 2).

To determine the therapeutic time window of JTE013 in experimental stroke, we assessed the oedema and infarct ratios after delayed administration of the S1PR2 antagonist. We found that JTE013 administration 4.5 h after ischaemia resulted in the same significant reduction of brain infarct and oedema ratios compared with 1.7 h or *S1pr2*^*−/−*^ groups (no statistically significant differences were found between the 4.5 h, 1.7 h and *S1pr2*^*−/−*^ groups, Supplementary Fig. 3), indicating that JTE013 was efficacious in reducing brain infarct and oedema when administered within 4.5 h after stroke. Administration of JTE013 at 7.5 h after the onset of ischaemia had reduced efficacy and did not lead to statistically significant lower infarct sizes compared with the vehicle-treated group, indicating that JTE013 is protective when given within 4.5 h of stroke onset.

Altogether our data indicate that genetic deletion of S1PR2 or administration of a S1PR2 antagonist in wild-type mice after reperfusion, dramatically decreased infarct size, total oedema and cell death in experimental stroke, resulting in improved neurological scores, indicating that S1PR2 could be a novel therapeutic target in stroke.

### Critical role of S1PR2 in cerebrovascular injury

**Figure 3:**
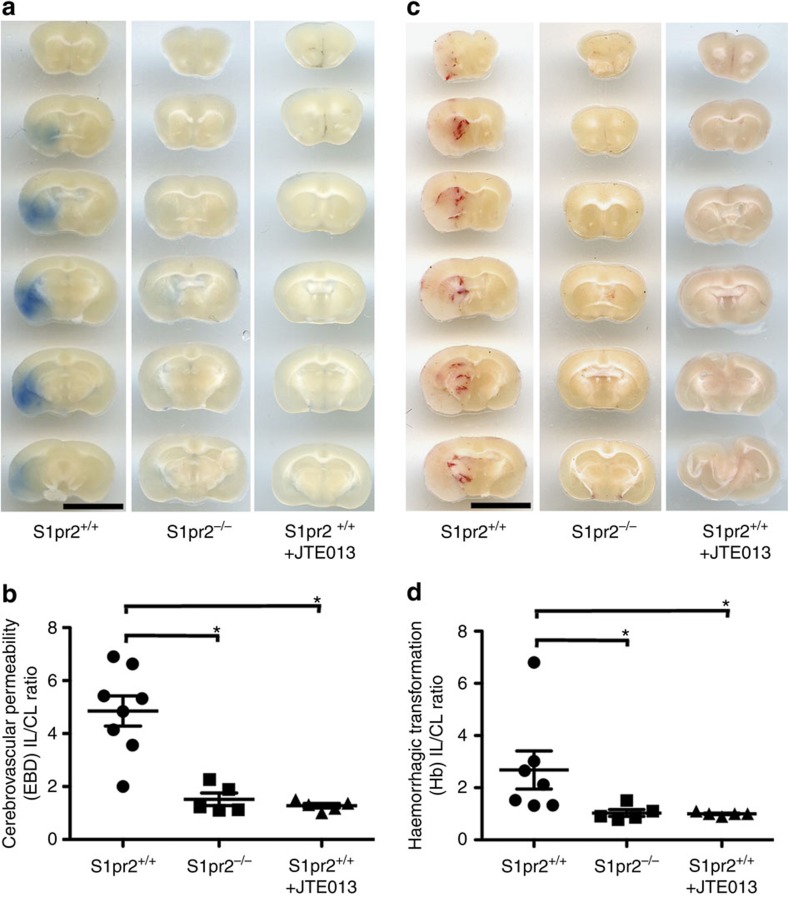
Critical role of S1PR2 in the disruption of cerebrovascular integrity after I/R injury. (**a**,**b**) Cerebrovascular permeability (vasogenic oedema) was determined by Evans blue dye (EBD) extravasation assay, 3 h after reperfusion in *S1pr2*^+/+^, *S1pr2*^−/−^ and *S1pr2*^+/+^receiving JTE013. (**a**) Brain coronal slices of representative animals. Scale bar, 5 mm. (**b**) Quantification of extravascular EBD. Ipsilateral/contralateral (IL/CL) ratios are shown. (**c**,**d**) Haemorrhagic transformation was assessed 24 h after reperfusion in *S1pr2*^+/+^, *S1pr2*^−/−^ and *S1pr2*^+/+^receiving JTE013. (**c**) Representative images are shown. Scale bar, 5 mm. (**d**) Quantification of extravascular haemoglobin (Hb) content. Values are ipsilateral/contralateral (IL/CL) ratios of micrograms of Hb. (**b**,**d**) The individual values and the mean±s.e.m. are shown, *n*=5–8 from four independent experiments. *P<0.05 (one-way ANOVA followed by Newman–Keuls).

During cerebral ischaemia, blood–brain barrier disruption starts early after the onset of ischaemia and increased cerebrovascular permeability can be detected as early as 3 h after reperfusion^25^,^26^. To investigate the role of S1PR2 in vasogenic oedema (increased cerebrovascular permeability), we conducted Evans blue dye (EBD) extravasation assays. As shown in Fig. 3a,b, *S1pr2*^−/−^ mice exhibited a dramatic decrease in EBD extravasation, 3 h after reperfusion, compared with their wild-type litter mates (ratio of EBD content in ipsilateral hemisphere/contralateral hemisphere was 1.6±0.31 in *S1pr2*^−/−^ mice versus 4.54±0.83 in wild-type). In addition, acute inhibition of S1PR2 signalling in wild-type mice, by JTE013 administration, potently blocked the induction of cerebrovascular permeability after I/R injury (ratio of EBD content 1.28±0.08, Fig. 3a,b).

Increasing clinical evidence indicates that areas of the brain with compromised blood–brain barrier at the early stages of ischaemic stroke often undergo haemorrhagic transformation following reperfusion^27^,^28^,^29^,^30^. To study the role of S1PR2 in the development of intracerebral haemorrhage after I/R injury, we used a model of spontaneous haemorrhagic transformation. Mice were subjected to 3 h of ischaemia to induce severe neurovascular injury. On this prolonged time of ischaemia, a significant loss of cerebrovascular integrity was observed 24 h after reperfusion in wild-type mice (Fig. 3c,d), as assessed by the presence of extravascular blood in the ischaemic hemisphere (448±96.4 mg haemoglobin) compared with the contralateral hemisphere (182±23.9 mg haemoglobin/hemisphere). The ipsilateral/contralateral ratio was 2.68±0.74 in wild-type mice (Fig. 3d). In sharp contrast, intracerebral haemorrhage was almost absent in *S1pr2*^−/−^ mice (ipsilateral/contralateral ratio 1.01±0.17, ∼80% inhibition). In addition, acute inhibition of S1PR2 by oral administration of JTE013, 10 min after reperfusion, dramatically inhibited the development of intracerebral haemorrhage (ipsilateral/contralateral ratio 1.04±0.05). The mortality rate at 24 h after a 3-h occlusion was 39% in vehicle-treated wild-type mice, 20% in *S1pr2*^−/−^ and 18.2% in wild-type mice treated with JTE013.

These data indicate the critical role of S1PR2 in the disruption of cerebrovascular integrity after I/R injury in the brain, suggesting that S1PR2 could be targeted in stroke patients at the time of reperfusion to reduce cerebrovascular permeability and haemorrhagic transformation.

### Critical role of S1PR2 in MMP-9 activation after stroke

**Figure 4:**
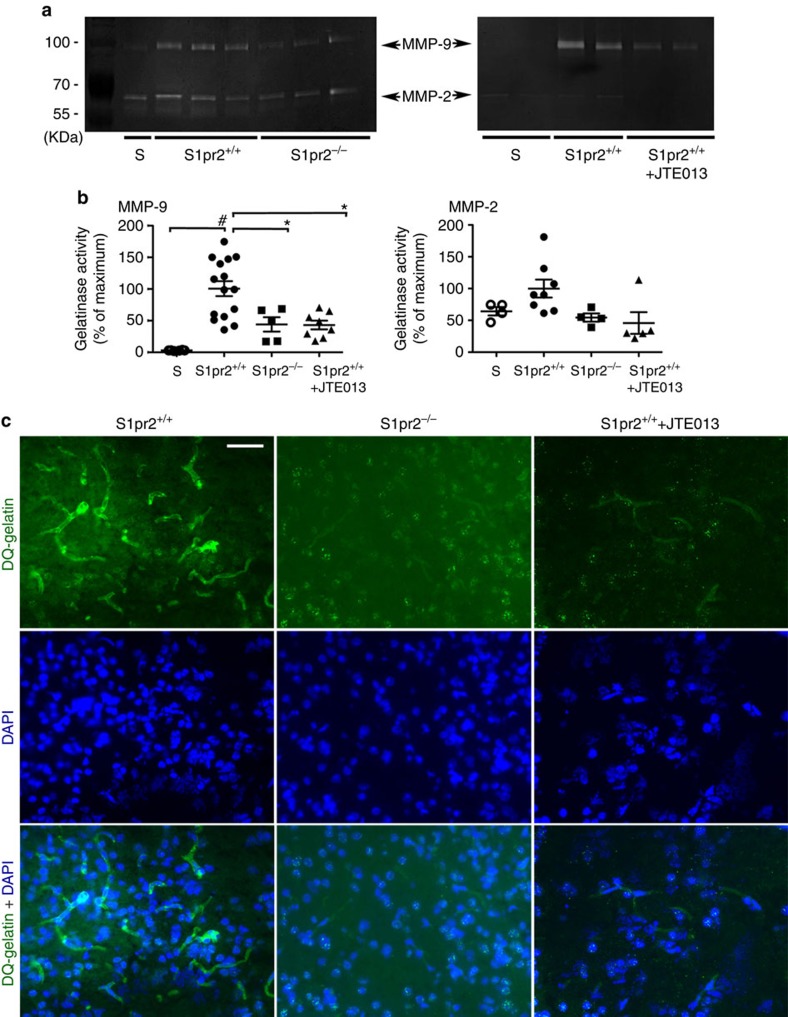
Blockade of S1PR2 results in decreased MMP-9 activity in ischaemic brains. (**a**) Representative zymographies showing MMP-9 and MMP-2 activity, 3 h after I/R in whole brain homogenates, in sham (S), vehicle-treated *S1pr2*^+/+^ (*S1pr2*^+/+^), vehicle-treated *S1pr2*^−/−^ (*S1pr2*^−/−^) and JTE013-treated *S1pr2*^+/+^ mice (*S1pr2*^+/+^+JTE013). Arrows indicate active MMP-9 and MMP-2. (**b**) Quantification of gelatinase activity by image analysis. Values are normalized by the average gelatinase activity in wild-type mice after I/R injury. *N*=4–15 from five independent experiments. #*P*<0.05 sham versus MCAO, *P≤0.05 vehicle-treated *S1pr2*^+/+^ versus JTE013-treated *S1pr2*^+/+^ or vehicle-treated *S1pr2*^−/−^ (one-way ANOVA followed by Newman–Keuls). (**c**) Representative pictures of the damaged area (at the level of bregma +1.2 to +0.8 mm, rostrally, as indicated in Fig .1g) of the *in situ* gelatinase assay in frozen brain sections (14 μm) using FITC DQ-gelatin are shown. Notice significantly lower gelatinase activity in brain microvessels in vehicle-treated *S1pr2*^−/−^ or JTE013-treated *S1pr2*^+/+^ mice compared with vehicle-treated wild-type, 3 h after reperfusion. *N*=5–6 per group, 4 independent experiments. Scale bar, 50 μm.

To elucidate the mechanisms of regulation of cerebrovascular integrity by S1PR2, we determined the activation of MMP-2 (gelatinase A) and MMP-9 (gelatinase B), also known as type IV collagenases, since they play a critical role in increased cerebrovascular permeability after I/R injury^31^,^32^. In wild-type animals, MMP-9 activity was significantly increased in brain lysates 3 h after I/R injury, compared with sham animals (Fig. 4a,b). Interestingly, *S1pr2*^−/−^ mice exhibited significantly decreased MMP-9 activity compared with wild-type (56±10% inhibition). Similarly, in JTE013-treated wild-type mice, MMP-9 activity was significantly lower compared with vehicle-treated wild-type mice (57±6.9% inhibition). In contrast, we did not detect a significant increase in MMP-2 activity in total brain lysates after I/R compared with sham animals or differences in MMP-2 activity between wild-type and *S1pr2*^−/−^ mice or JTE013-treated mice 3 h after reperfusion. These data indicate that S1PR2 plays a critical role in the induction of MMP-9 activity in brain lysates after I/R injury. *In situ* zymography assays revealed a dramatic increase in gelatinase activity both in cerebral microvessels and in brain parenchyma in the damaged area in vehicle-treated wild-type mice, 3 h after reperfusion (Fig. 4c) (no signal was detected in sham animals or in the contralateral hemisphere). Interestingly, *S1pr2*^−/−^ and JTE013-treated *S1pr2*^*+/+*^ mice exhibited much lower gelatinase activity in cerebral microvessels, consistent with the decreased cerebrovascular integrity observed in the EBD cerebrovascular permeability assay. Representative pictures of the damaged area at the level of bregma +1 mm (rostrally) are shown, as indicated in Fig. 1g. Altogether these data indicate that S1PR2 plays a critical role in MMP-9 activation and the induction of gelatinase activity in cerebral microvessels after I/R injury in experimental stroke.

### Detection of S1PR2 in cerebral microvessels after I/R injury

**Figure 5:**
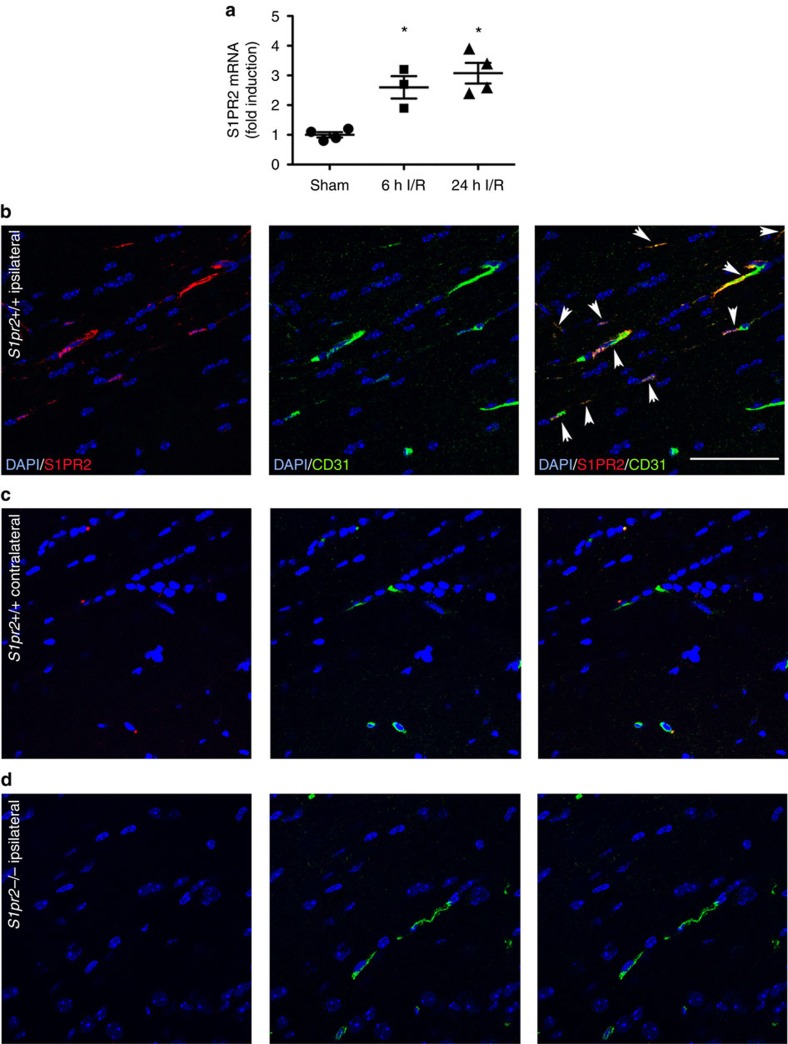
Upregulation of S1PR2 in brain microvessels after I/R injury. (**a**) S1PR2 mRNA levels in the ipsilateral hemisphere of brain homogenates from sham animals and MCAO animals, 6 h (6 h I/R) or 24 h (24 h I/R) after tMCAO. The fold induction of S1PR2 mRNA levels (normalized by 18S RNA) versus sham is shown. *P<0.05 (one-way ANOVA followed by Newman–Keuls). (**b**–**d**) S1PR2 (red channel) and CD31 (green channel) immunofluorescence analysis in brain sections from the ipsilateral (**b**) or contralateral (**c**) hemisphere of wild-type mice or ipsilateral hemisphere of *S1pr2*^*−/−*^ mice (**d**) 6 h after tMCAO. Representative pictures of the corpus callosum at the level of bregma +1.2 to +0.8 mm, rostrally, as indicated in Fig. 1g, are shown (*n*=5–6 per group). S1PR2 was detected only in cerebral microvessels in the damaged area (ipsilateral hemisphere), (**b**) but not in the contralateral hemisphere (**c**), the ipsilateral hemisphere of *S1pr2*^*−/−*^ mice (**d**), or in sham animals (not shown). Arrows indicate the S1PR2 and CD31 positive areas. Scale bar, 50 μm.

We next aimed to assess the expression of S1PR2 in the mouse brain after I/R injury. In total brain lysates, we found that S1PR2 mRNA basal levels were low (0.37±0.03 mRNA copies/10^6^ 18S mRNA) but were significantly increased in the ischaemic hemisphere after I/R injury (2.6±0.37- and 3.1±0.35-fold at 6 and 24 h, respectively, Fig. 5a). Consistent with this mRNA data, we detected S1PR2 protein 6 h after I/R injury by immunofluorescence analysis only in the ischaemic hemisphere of wild type mice (Fig. 5b). Representative pictures of the damaged area (corpus callosum and striatum) at the level of bregma +1 mm (as indicated in Fig. 1g) are shown (Fig. 5b and Supplementary Fig. 4a, respectively). S1PR2 positivity colocalized with CD31 staining (Fig. 5b and Supplementary Fig. 4a). No S1PR2 immunopositivity was detected in the contralateral hemisphere of wild type mice 6 h after I/R injury (Fig. 5c and Supplementary Fig. 4b), in the ispilateral hemisphere of *S1pr2*^*−/−*^ mice (Fig. 5d and Supplementary Fig. 4c) or in sham animals. These data indicate that S1PR2 upregulation in cerebral microvessels is an early event after I/R injury.

### S1PR2 is critical for brain endothelial cell activation

**Figure 6:**
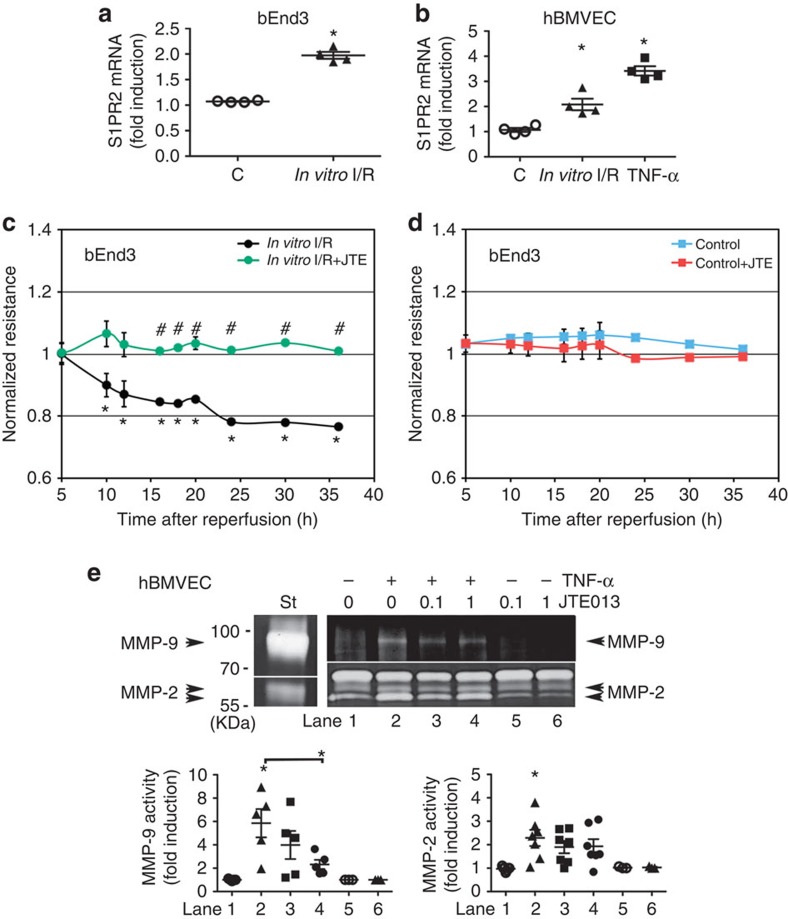
Critical role of S1PR2 in brain endothelial cell permeability and MMP-9 activation. (**a**,**b**) Induction of S1PR2 mRNA in bEnd.3 and hBMVEC after *in vitro* I/R injury or activation with TNF-α. Brain endothelial cells were subjected to (**a**) 6 h of OGD followed by *in vitro* reperfusion or (**b**) activation with TNF-α (5 ng ml^−1^). 6 h later, RNA was isolated and S1PR2 mRNA levels were quantified by qRT–PCR analysis. Fold induction of S1PR2 mRNA (normalized by 18S RNA) versus control (C) is shown. *P≤0.05 *in vitro* I/R or TNF-α versus control (Student’s *t*-test). Values are mean±s.e.m., *n*=4. (**c**,**d**) S1PR2 inhibition by JTE013 prevented the decrease in monolayer resistance in bEnd.3 cells after *in vitro* I/R injury. (**c**) *In vitro* I/R. (**d**) Control (normoxia). Values are mean±s.e.m. *n*=3–7 from three independent experiments. *P≤0.05 *in vitro* I/R versus control (normoxia, right panel), #*P*≤0.05 JTE013-treated *in vitro* I/R versus vehicle-treated *in vitro* I/R (Student’s *t*-test). Black: *in vitro* I/R+vehicle, green: *in vitro* I/R+JTE013, blue: control (normoxia)+vehicle, red: control (normoxia)+JTE013. (**e**) MMP-9 and MMP-2 activity in human brain microvascular endothelial cell (hBMVEC) supernatants after treatment with 5 ng ml^−1^ TNF-α in the presence or absence of the S1PR2 antagonist JTE013 (0.1 or 1 μM) for 24 h. Values are mean±s.e.m. *n*=4–5 from four independent experiments. A representative zymography is shown. *P≤0.05 TNF-α versus control, and when indicated TNF-α+vehicle versus TNF-α+JTE013-treated (one-way ANOVA followed by Newman–Keuls). St: active MMP-2 and MMP-9 standards. Arrows indicate active MMP-9 and MMP-2.

Given our *in vivo* findings in mice, we next aimed to study the role of S1PR2 in the regulation of the responses of brain endothelial cells to I/R injury *in vitro*. We used the mouse brain microvascular endothelial cell line, bEnd3 (ref. 33), and hBMVEC. To mimic *in vitro* the metabolic and inflammatory stress of I/R injury, we conducted oxygen and glucose deprivation (OGD) experiments *(in vitro* I/R), as well as TNF-α activation studies. *In vitro* I/R injury or TNF-α activation resulted in upregulation of S1PR2 message (2- and 3.5-fold, respectively) both in bEnd3 and hBMVEC (Fig. 6a,b). To study the role of S1PR2 in brain endothelial cell permeability, bEnd3 cells were exposed to hypoxia/aglycemia (OGD) for 6 h. Then, we measured changes in endothelial resistance on *in vitro* reperfusion, using the electrical cell-substrate impedance sensing system (ECIS) (Applied BioPhysics, NY)^34^. As shown in Fig. 6c, in vehicle-treated cells, endothelial resistance starts to decrease 10 h after *in vitro* reperfusion and remains lower compared with control (normoxia, Fig. 6d). However, inhibition of S1PR2 signalling by JTE013 abrogated the decrease in monolayer resistance observed after *in vitro* I/R injury (Fig. 6c), indicating the critical role of S1PR2 in the induction of brain endothelial cell monolayer permeability after *in vitro* I/R injury. JTE013 treatment did not affect endothelial resistance under normoxic conditions (Fig. 6d). These experiments were conducted in the presence of serum, which contains S1P. In addition, since endothelial cells exhibit high levels of SPHK activity^19^ and are one of the main sources of plasma S1P^14^ it is possible that endothelial cell-derived S1P activates S1PR in an autocrine way, as we have previously described^20^.

Given that our *in vitro* model demonstrated similar S1PR2-mediated vascular responses to I/R injury as to those observed *in vivo*, we next investigated the role of S1PR2 in MMP activation in brain endothelial cells. Activation of human brain endothelial cells with TNF-α (5 ng ml^−1^) for 24 h resulted in increased MMP-9 activity in the conditioned media compared with vehicle-treated cells (5.8±1.2-fold induction, Fig. 6e). In addition, TNF-α treatment induced the cleavage of pro-MMP-2 (72 kDa) into the 67 and 64 kDa cleaved active forms (2.3±0.3-fold versus control). Blockade of S1PR2 signalling by JTE013 (1 μM)^20^ significantly decreased MMP-9 activity in the conditioned media, while it did not significantly change the levels of the cleaved active MMP-2 forms (Fig. 6e). Consistent with the MMP activity data in endothelial conditioned media, we found that MMP-9 mRNA levels in brain endothelial cells were low under basal conditions and upregulated by TNF-α (Supplementary Fig. 5a), while MMP-2 mRNA levels were constitutively high and not affected by TNF-α (Supplementary Fig. 5b). Also, JTE013 partially inhibited the induction of MMP-9 mRNA by TNF-α, while it did not affect MMP-2 mRNA levels. These data are consistent with the critical role of S1PR2 in endothelial activation^21^. Using a gain-of-function approach, we found that upregulation of S1PR2 levels by adenoviral transduction in hBMVEC resulted in increased levels of MMP-9 mRNA and MMP-9 activity (Supplementary Fig. 5c–e) compared with control adenovirus-transduced cells (β-galactosidase). These data indicate that S1PR2 upregulation in brain endothelial cells is sufficient for the induction of MMP-9 mRNA and MMP-9 activity in endothelial cell-conditioned media. Altogether our *in vitro* data indicate that S1PR2 plays a critical role in the induction of brain endothelial cell permeability after I/R injury and activation of MMP-9.

### S1PR2 is dispensable for neural and glial responses *in vitro*

**Figure 7:**
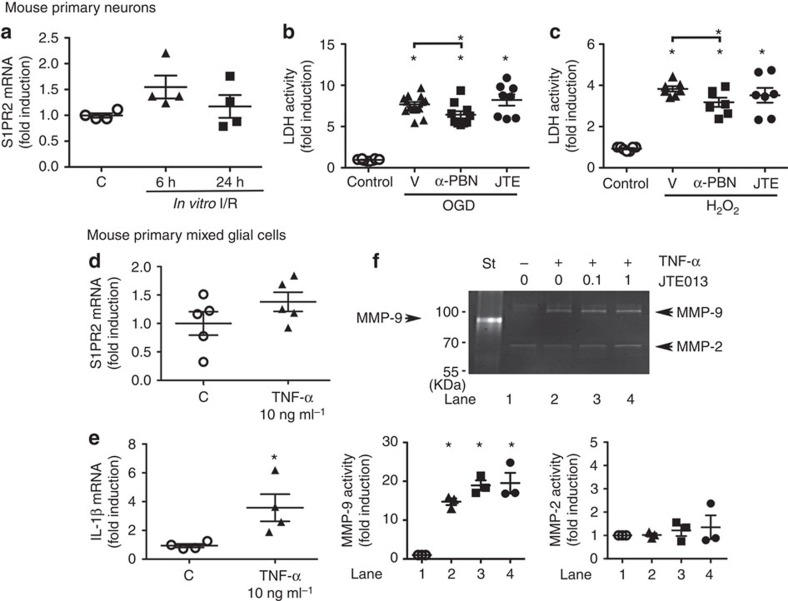
Role of S1PR2 in the responses of neurons and glial cells to injury. Mouse primary neurons (**a**–**c**) and mouse primary mixed glial cells (**d**,**e**) were isolated and cultured as described in Methods. (**a**) Mouse primary neurons were subjected to 4 h of OGD. 6 or 24 h after *in vitro* reperfusion, RNA was isolated and S1PR2 mRNA copy number was calculated by qRT–PCR analysis and normalized by 10^6^ copies of 18S RNA. Fold induction versus control (C; normoxia, normoglycemia) is plotted. (**b**) Mouse primary neurons were subjected to 4 h of OGD. On *in vitro* reperfusion, cells were treated with vehicle (V), α-PBN (1 mM) or JTE013 (1 μM) for 24 h. Neuronal cell death was assessed by measuring LDH activity in the supernatants. (**c**) Primary neurons were treated with H_2_O_2_ (30 μM) for 24 h in the presence or absence of 1 mM α-PBN or 1 μM JTE 013 (JTE) and LDH activity was measured. Control: no H_2_O_2_. V: vehicle. (**d**,**e**) Mouse primary mixed glial cells were activated with 5 ng ml^−1^ TNF-α to mimic the pro-inflammatory environment of I/R injury. 24 h later, S1PR2 (**d**) and IL-1β (**e**) mRNA levels were assessed by qRT–PCR analysis. (**f**) Mouse primary mixed glial cells were activated with 5 ng ml^−1^ TNF-α in the presence or absence (vehicle) of JTE013 at the doses indicated (μM). MMP-9 and MMP-2 activity in the conditioned media was assessed by gelatin zymography. Values are mean±s.e.m. *n*=3 from three independent experiments. A representative zymography is shown. *P≤0.05 TNF-α versus control, and when indicated TNF-α versus TNF-α+JTE013 treated (one-way ANOVA followed by Newman–Keuls). St: active MMP-9 standard. (**a**–**e**) Fold induction versus control is plotted.

To assess the role of S1PR2 in the regulation of neuronal and glial responses to ischaemic and inflammatory injury, we isolated and cultured mouse cortical primary neurons from embryos (E 17.5) and mixed glial cells from pups (post natal day 2). *In vitro* I/R injury (4 h of OGD followed by *in vitro* reperfusion) did not significantly change the levels of S1PR2 mRNA in mouse cortical primary neurons (Fig. 7a). In addition, blockade of S1PR2 signalling by JTE013 did not inhibit neuronal cell death induced by *in vitro* I/R injury, assessed by lactate dehydrogenase activity (LDH) activity in neuronal supernatants (Fig. 7b), while α-phenyl-tert-butyl nitrone α-(PBN), a neuroprotective spin trap agent that reacts with and trap free radicals^35^, did inhibit. Similarly, neuronal cell death induced by H_2_O_2_ was not affected by JTE013 but it was inhibited by α-PBN (Fig. 7c). In mouse primary mixed glial cells, activation with TNF-α did not significantly increase the levels of S1PR2 mRNA (Fig. 7d), while it significantly induced the expression of other pro-inflammatory molecules, such as interleukin-1β (Fig. 7e). In addition, we found that JTE013 did not affect the inflammatory responses of mouse primary mixed glial cells. As shown in Fig. 7f, the induction of MMP-9 activity by TNF-α in glial cell supernatants was not inhibited by the S1PR2 antagonist, JTE013. These data indicate that S1PR2 is not critical for the regulation of neuronal and glial responses to ischaemic and inflammatory injury *in vitro*. Altogether our *in vitro* data with brain endothelial cells, neurons and glial cells indicate that S1PR2 plays a critical role in the responses of brain endothelial cells to I/R injury.

### Expression of S1PR2 in human brain samples

**Figure 8:**
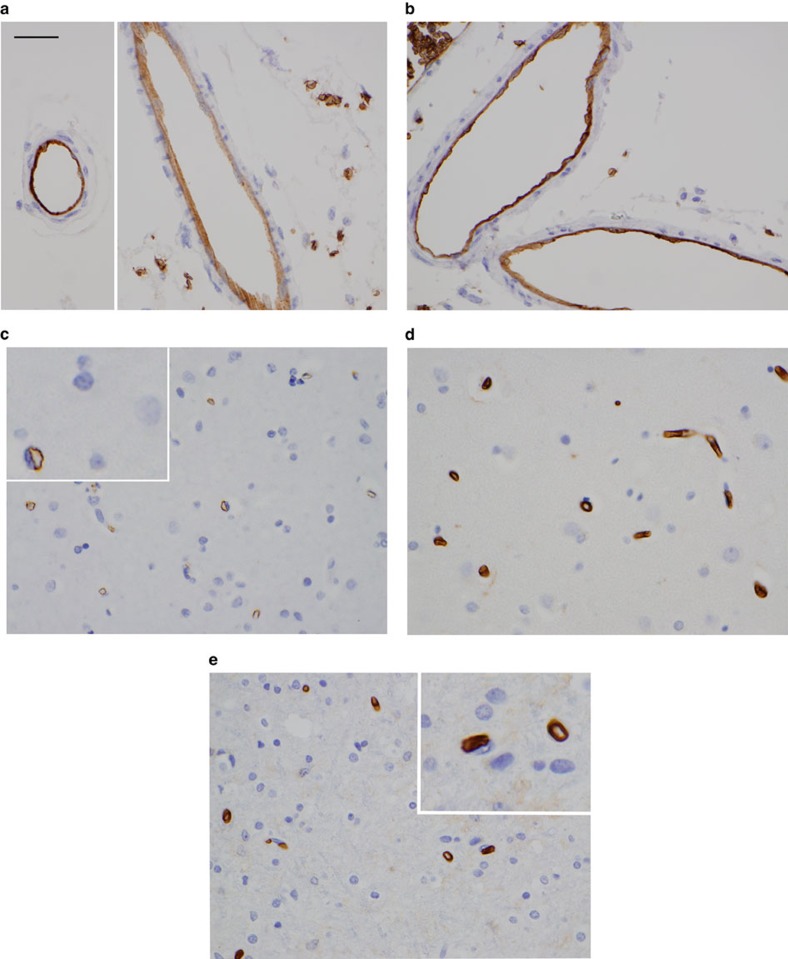
Immunohistochemical analysis of S1PR2 in human brain. S1PR2 was detected in endothelial cells of pial vessels and microvessels in autopsy specimens. Representative images are shown. (**a–e**) Cases 1–5. Pictures were taken at × 60 magnification. Scale bar, 30 μm.

Given our *in vivo* and *in vitro* findings, to assess their potential pathophysiological relevance in humans we performed immunohistochemical analysis of S1PR2 in human brain autopsy samples. The specificity of the S1PR2 antibody was determined as we have previously described^36^: S1PR2 immunohistochemistry showed membranous and cytoplasmic staining only in S1PR2-transfected 293T cells and not in S1PR1-transfected or pcDNA3.1-transfected cells (Supplementary Fig. 6). Immunohistochemical analysis of human brain samples revealed S1PR2 positivity in the cerebrovascular endothelium (Fig. 8, representative pictures). We found heterogeneous S1PR2 expression in the endothelium of pial vessels and microvessels throughout the brain in the five brain samples analysed. The limited clinical data available for these patients (summarized in Supplementary Table 1) revealed various hypoxic/ischaemic states including stroke, respiratory failure or sickle cell vaso-occlusive crisis. These data indicate that S1PR2 can be detected in the human cerebrovascular endothelium.

## Discussion

In this study, we have identified the critical role of S1PR2 in the disruption of neurovascular integrity after I/R injury. We found that genetic deletion or pharmacological inhibition of S1PR2 promoted cerebrovascular integrity in a mouse model of transient cerebral ischaemia (tMCAO), resulting in decreased neuronal death and improved neurological scores. Inhibition of S1PR2 potently blocked the development of cerebral oedema and spontaneous haemorrhagic transformation in experimental stroke. We also found that S1PR2 signal is detected in cerebral microvessels in mice in the early stages after I/R injury. In addition, our *in vivo* and *in vitro* data provide mechanistic insights into the regulation of cerebrovascular permeability by S1PR2. *In vivo*, we found that genetic deletion or pharmacological inhibition of S1PR2 results in decreased MMP-9 activity in whole brain lysates and gelatinase (MMP-2/9) activity in cerebral microvessels, which is consistent with the decreased cerebrovascular permeability and haemorrhagic transformation observed in *S1pr2*^−/−^ mice or wild-type mice treated with JTE013 compared with vehicle-treated wild-type mice. Our *in vitro* studies indicate that S1PR2 plays a critical role in the responses of brain endothelial cells to I/R injury (that is, permeability and MMP-9 activation) but not neurons or glial cells. In experimental stroke, MMP have been shown to play a critical role in the blood–brain barrier disruption and haemorrhagic transformation^10^,^37^,^38^. More specifically, MMP-9 levels and activity are increased early during cerebral ischaemia^38^ and mice lacking MMP-9 (ref. 8), but not MMP-2 (ref. 39), are protected in stroke models. Interestingly, in stroke patients, increased plasma levels of MMP-9, but not other MMP, have been associated with increased risk of developing haemorrhagic transformation^40^,^41^. Although endothelial cells, astrocytes, neurons and immune cells can upregulate MMP-9, several studies point to the cerebrovascular endothelium as an important source of MMP-9 activity in the early stages of ischaemia contributing to the early disruption of the blood–brain barrier^8^,^31^,^42^. Altogether, our *in vivo* and *in vitro* data point to the activation of MMP-9 in the endothelium as a mechanism of induction of cerebrovascular permeability by S1PR2 after I/R injury.

**Figure 9:**
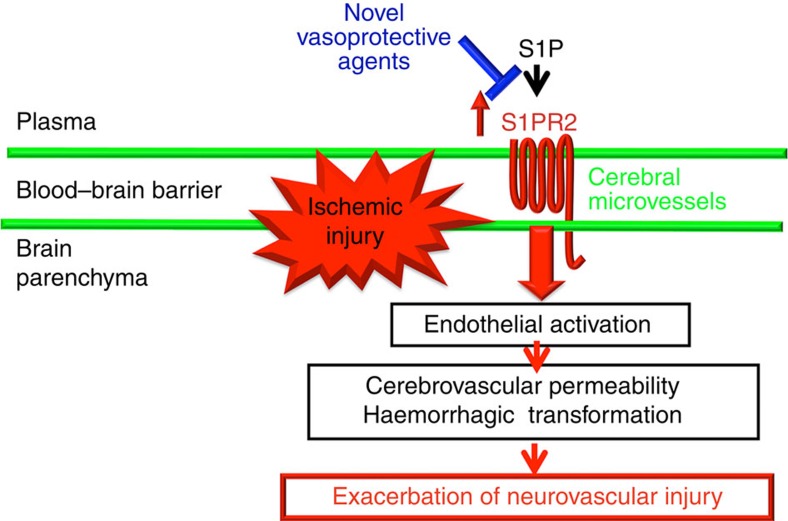
Critical role of S1PR2 in the disruption of cerebrovascular integrity in stroke. Diagram summarizing our findings and the potential of S1PR2 antagonists as novel neurovascular protective therapies in stroke.

Our findings are of potential translational relevance since the use and effectiveness of current stroke reperfusion therapies are limited by subsequent development of cerebrovascular complications of reperfusion injury (that is, vasogenic oedema and haemorrhagic transformation). Increased cerebrovascular permeability plays a critical role in the pathophysiology of stroke and I/R injury by allowing the entrance of neurotoxic plasma components and blood cells into the brain parenchyma, which compromises synaptic and neuronal function and by increasing intracerebral pressure, with the risk of brain herniation and/or compression of cerebral vessels, further compromising blood flow to the brain. In animal models of cerebral ischaemia, blood–brain barrier disruption occurs early and continues after reperfusion^25^,^26^. In stroke patients, early blood–brain barrier disruption is associated with haemorrhagic transformation and poor clinical outcomes^29^. Haemorrhagic transformation is a serious complication that limits the effectiveness and use of current stroke reperfusion therapies (thrombolytic and mechanical recanalization)^43^, but it may also spontaneously develop as part of the natural evolution of ischaemic brain injury^44^. In this study, we found that S1PR2 inhibition potently blocked the development of spontaneous haemorrhagic transformation in experimental stroke, which was observed after a more severe ischaemic injury. In addition, our findings that *S1pr2*-deficient mice or wild-type mice, treated with the S1PR2 antagonist JTE013, exhibited a dramatic decrease in cerebral oedema also raise the intriguing possibility that S1PR2 targeting could be potentially beneficial to prevent the development of malignant MCA syndrome, in which rapid brain oedema following a large MCA infarct leads to herniation and high mortality rates (80%)^45^, particularly in younger stroke patients^46^,^47^. Current therapeutic options to control cerebral oedema in stroke aim at decreasing intracerebral pressure by osmotherapy^48^, hyperventilation, administration of diuretics, induction of coma^49^ or hypothermia, among others^50^. Although most of these conservative treatments have failed to improve mortality and disability^49^,^50^, they are still being used due to the lack of alternative therapies. Only surgical decompression has been shown to be effective but only in younger patients due to the risk associated with the surgery^51^. In our study, we found that S1PR2 expression is detected in brain microvessels after stroke, which together with our *in vitro* data with brain endothelial cells, neurons and glial cells, indicate that S1PR2 plays a critical role in the disruption of cerebrovascular integrity. We also found that the S1PR2 antagonist, JTE013, is protective when given within 4.5 h of stroke onset (therapeutic time window). These findings have a high translational relevance and could potentially develop into novel therapies, especially with the increasing clinical use of thrombolytic or mechanical reperfusion therapies in stroke patients. In contrast to the efforts made in the development of neuroprotective drugs, very few studies have focused on the development of agents that promote cerebrovascular integrity. Our findings suggest that administration of vasoprotective agents (for example, a S1PR2 antagonist) at the time of reperfusion could be a novel strategy to diminish cerebrovascular complications of reperfusion injury, ultimately resulting in less neurovascular injury and improved stroke outcomes^52^,^53^,^54^ (Fig. 9).

The pathophysiological relevance of our *in vivo* findings in mice is strengthened by the fact that we detected the expression of S1PR2 in the cerebrovascular endothelium in human brain samples. More specifically, we have detected S1PR2 expression in the cerebrovascular endothelium of pial vessels and microvessels in human tissue. Our human study is limited by the fact that only autopsy material was available and also by the low number of cases. A series of a larger number of cases, and ideally biopsy specimens or autopsy material collected within few hours after death, would be required to more definitively characterize the regulation of S1PR2 expression in the cerebrovascular endothelium after ischaemic/hypoxic/inflammatory injury in the human brain. Nevertheless, we can conclude that S1PR2 is detected in the human cerebrovascular endothelium, which is consistent with a recent report by Cruz-Orengo *et al.*^55^ in which they found that S1PR2 is upregulated in cerebellar and spinal cord microvessels in multiple sclerosis lesions. Altogether our data suggest that S1PR2 is a novel and attractive target, which deserves further study in terms of the potential for pharmacological blockade to promote cerebrovascular integrity in humans.

S1PR are becoming attractive candidates for drug discovery. The most well-characterized S1PR, S1PR subtype 1, was originally cloned from human endothelial cells^56^, and plays a critical role in vascular integrity^18^,^19^ and lymphocyte trafficking and egress from lymphoid organs^22^,^57^,^58^. In fact, the recently approved drug by FDA for the treatment of multiple sclerosis, FTY720, a potent immunosuppressor, is an agonist of S1PR1, 3, 4 and 5. In recent studies, FTY720 has been shown to confer neuroprotection in animal models of stroke^59^,^60^ via its immunosuppressive effects^60^ but not by direct neuroprotection^59^,^60^. However, FTY720 treatment is associated with increased risk of infection in multiple sclerosis patients^61^. Increasing clinical and preclinical evidence indicates that stroke-induced immunosuppression is the cause of increased vulnerability of stroke patients to infection^62^,^63^, a leading cause of prolonged intensive care unit stays and increased mortality in stroke patients. Therefore, further research is still needed before considering immunomodulation (for example, FTY720) as a therapeutic alternative for human stroke. In contrast to these previous reports^59^,^60^, our study is focused on a different S1P receptor, S1PR2, which is not targeted by FTY720. Our recent work has identified endothelial S1PR2 as a robust mediator of vascular permeability and acute vascular injury in several organs in sepsis models^21^. In addition, we found no differences in immune cell trafficking between wild-type and *S1pr2* null mice^21^. Therefore, targeting of this novel pathway would have a potential advantage compared with targeting S1PR1 since it could lead to neurovascular protection without compromising immune function, which could be especially beneficial for stroke patients.

Recent studies from our lab indicate that S1PR2 plays a role in endothelial activation (permeability and inflammation) during acute inflammatory vascular injury^20^,^21^. On activation with pro-inflammatory stimuli, SPHK-1 activity, the rate-limiting enzyme in S1P generation, is increased in endothelial cells^64^ and S1P is released. S1PR2 expression is also induced in endothelial cells during inflammation, while the levels of the other receptors do not change significantly^21^. Our *in vitro* data is consistent with a model in which the binding of endothelial-derived or serum-derived S1P to S1PR2 is blocked by JTE013, resulting in inhibition of brain endothelial cell activation (for example, permeability and MMP-9 activity) after *in vitro* I/R injury or inflammatory challenge. The induction of MMP-9 by S1PR2 likely involves the activation of the Rho–ROCK–NFκB and the stress-activated protein kinase pathways^12^, two pro-permeability and pro-inflammatory pathways that are activated by S1PR2 (ref. 21). Although S1PR2 is emerging as an important mediator of endothelial dysfunction in septic/inflammatory and ischaemic vascular injury^20^,^21^, it has been shown to play a protective role in anaphylactic shock via inhibition of endothelial nitric oxide synthase^65^,^66^. Therefore, the effect of S1PR2 activation in terms of exacerbating or ameliorating disease appears to depend on the underlying pathophysiological setting. A better understanding of the molecular mechanisms involved in the regulation of S1PR2 expression in different vascular beds and cell lineages, as well as its downstream molecular mediators is required to determine appropriate targeting strategies of the S1PR2 pathway in various pathophysiological settings.

In conclusion, our data indicate that S1PR2 plays a critical role in the disruption of neurovascular integrity after I/R injury and MMP-9 activation in brain endothelial cells. These findings reveal the novel role of S1PR2 in the pathophysiology of cerebrovascular complications of I/R injury in experimental stroke. Altogether our *in vitro* and *in vivo* data in mice and humans point to S1PR2 as an attractive therapeutic target for cerebrovascular protection in stroke and other instances of hypoxic or inflammatory injury in cerebral blood vessels.

## Methods

### Mice

Mice with targeted disruption of the *S1pr2* gene^23^ were maintained on a mixed C57BL/6;129Sv genetic background (five times backcrossed to C57BL/6). Experiments on knockout mice were performed with appropriate wild-type littermate controls. All animal studies were approved by the Beth Israel Deaconess Medical Center Institutional Animal Care and Use Committee.

### tMCAO and treatments

MCAO was performed using wild-type and knockout male mice (25–30 g) as previously described^24^,^67^. In brief, surgery was performed using a dissecting surgical microscope. Temperature was maintained at 36.5–37 °C, controlled by a thermostatic blanket (TC-1000 Temperature Controller, CWE, USA) throughout the procedure. Mice were anesthetized with isoflurane, delivered by facemask in O_2_-enriched air. Blood flow in the MCA territory was monitored throughout the procedure using by a laser Doppler probe (PeriFlux system 5000, Ardmore, PA, USA), placed on the skull directly over the territory of the left MCA perfusion area. A midline incision was made in the neck (1.5-cm long), the common carotid and external carotid arteries were dissected from the adjacent tissue. After occlusion of the common carotid by a microclip, the left external carotid was ligated, coagulated and cut. Nylon monofilament (6-0) with a round tip (∼0.22 mm) was introduced gently up to ∼8.5–9 mm towards the origin of the MCA. The successful occlusion was verified by laser Doppler flowmetry (75–90% blood flow decrease of the baseline). After the successful occlusion, the nylon monofilament was secured in place by ligation. About 90 or 180 min after occlusion, the suture was withdrawn to allow reperfusion. Animals were excluded from the experimental group only if the CBF did not decrease by 75–90% during occlusion or it did not recover up to 70% of baseline within 10 min after the start of reperfusion (a total of eight animals were excluded from the study). About 10 min after reperfusion, animals were randomly assigned to the treatment groups: JTE013 (10 or 30 mg kg^−1^ by gavage)^21^ or vehicle (2% 2-hydroxypropyl β-cyclodextrin in saline). To determine the therapeutic time window of JTE013 administration, vehicle or 30 mg kg^−1^ JTE013 were administered 7.5, 4.5 or 1.7 h (10 min after reperfusion) after the onset of ischaemia. For the 3-h time point assays (for example, determination of cerebrovascular permeability), JTE013 or vehicle was administered just before occlusion. After the surgery, all animals were maintained in a small animal heated recovery chamber (IMS Vetcare Chamber Recovery Unit, Harvard Apparatus, Holliston, MA).

### TTC staining and determination of infarct and oedema ratios

Staining with 2,3,5-triphenyltetrazolium chloride (TTC) was carried out by an independent investigator to identify viable and nonviable brain tissue. Because TTC is metabolized by intact mitochondria, the red areas indicate the living tissue. Brains were harvested, sectioned into 1-mm-thick slices and incubated in a 1% solution of TTC in phosphate-buffered saline at 37 °C for 5–10 min. Scanned images were used to calculate infarct and oedema ratios using image analysis software (Image J, the National Institutes of Health, Bethesda, MD). Infarct ratios were obtained after normalization by the contralateral (non-ischaemic) hemisphere and corrected for oedema as previously described 67. Briefly, the infarct ratio (*I*), corrected for oedema, was calculated by using the following equation: *I*=(*X*−*S*)/*Z*, where *X* is the area of infarct (mm^2^), *S* is brain swelling (mm^2^), *S*=*Y*−*Z*, *Y* is the area of the infarcted (ipsilateral) hemisphere slice (mm^2^) and *Z* is the area of the non-infarcted (contralateral) hemisphere slices (mm^2^). The oedema ratio was calculated with the following formula: *E*=(*Y*−*Z*)/*Z*.

### Neurological score evaluation

Neurologic examinations were carried out by an independent investigator 24 h after tMCAO. Neurological scores were calculated as followed: 0: no deficit, 1: forelimb weakness and torso turning to the ipsilateral side when held by tail, 2: circling to affected side when held by tail on the bench, 3: spontaneous circling to the affected side or unable to bear weight on the affected side and 4: no spontaneous locomotor activity or barrel rolling^67^.

### *In situ* cell death detection

DNA strand breakage during apoptosis after ischaemic brain damage was assessed by a TUNEL method using the In Situ Cell Death Detection Kit, TMR red (Roche Applied Science, Manheim, Germany), according to the manufacturer's instructions. Briefly, brain sections (30 μm) were permeabilized (0.1% Triton X-100, 0.1% sodium citrate solution) for 5 min on ice and then incubated with TUNEL reaction mixture (1:10, enzyme solution/label solution) for 1 h at 37 °C. DAPI (4′,6-diamidino-2-phenylindole) staining was used as a counter staining. Images were captured with the Axio Imager A1 microscope, using AxioCam MRc camera and the AxioVision 4.8 programme (Carl Zeiss Inc.) (original magnification, × 40). For quantification, the number of TUNEL-positive cells/field from three different fields in the damaged area per mouse (bregma +1.2 to +0.8 mm, rostrally) were counted and the average values were plotted.

### EBD extravasation assay

To assess cerebrovascular permeability, 1 h after reperfusion, a 2% EBD solution (4 mL kg^−1^) was injected into the lateral tail vein. EBD binds to plasma albumin and it can be quantified in the extravascular compartment if there is increased vascular permeability. After 2 h, animals were anesthetized and perfused through the left ventricle with ice-cold PBS to remove intravascular EBD. Brains were harvested, sliced and scanned. Then, the hemispheres were separated and mechanically homogenized in 50% trichloroacetic solution. After centrifugation (20,000*g* for 20 min), supernatants were diluted 1:3 with ethanol. The amount of extravascular EBD was calculated by measuring the fluorescence (620 excitation, 680 emission). Ipsilateral/contraleral ratios were calculated and plotted.

### Quantification of extravascular haemoglobin

To assess haemorrhagic transformation, 24 h after a 3-h occlusion period, mice were anesthetized and perfused through the left ventricle with a 5 mM EDTA-saline solution. Brains were harvested, sliced and scanned. Then, the ipsilateral and contralateral hemispheres were separated and homogenized in PBS. After sonication, the homogenates were centrifuged at 13,000 r.p.m. for 30 min at 4 °C and extravascular haemoglobin was quantified using the QuantiChrom TM Hemoglobin Assay Kit (BioAssay Systems, Hayward, CA) following manufacturer’s instructions. The amount of haemoglobin in brain homogenates was calculated using a standard curve. Ipsilateral/contraleral ratios were calculated and plotted.

### Determination of gelatinase activity in brain lysates

Three hours after reperfusion mice were perfused through the left ventricle with ice*-*cold PBS and brains were removed and frozen in liquid nitrogen. Brain tissue was homogenized in lysis buffer (50 mmol l^−1^ Tris-HCL pH 7.6, 150 mmol l^−1^ NaCl, 5 mmol l^−1^ CaCl2, 0.05% Brij-35, 0.02% NaN3 and 1% Triton X-100) and centrifuged at 13,000 r.p.m. for 20 min^10^. About 0.8 mg protein in 500 μl lysis buffer was incubated with 40 μl (bead volume) of gelatin-Sepharose 4B beads (GE Healthcare, Piscataway, NJ, USA) for 1 h at 4 °C with gentle rotation to pull down MMP from brain homogenates. The beads were washed three times with lysis buffer and were resuspended in 30 μl of elution buffer (10% dimethylsulfoxide in lysis buffer) for 30 min. The supernatants were collected and then each sample was mixed with equal amounts of SDS sample buffer (Invitrogen Life Technologies, Carlsbad, CA, USA). The samples were loaded on 10% SDS–polyacrylamide electrophoresis gels with 0.1% porcine skin gelatin as a substrate (Invitrogen Life Technologies). After electrophoresis, the gels were incubated with renaturing buffer (2.5% Triton X-100 in H_2_O) for 30 min to remove the SDS and then incubated for 24 h (for MMP-2) or 48 h (for MMP-9) at 37 °C with developing buffer (Invitrogen Life Technologies). After incubation, gels were stained for 60 min in Coomassie blue R-250 (0.25% Coomassie brilliant blue R-250, 50% methanol, 10% acetic acid) and de-stained in 40% methanol and 10% acetic acid until clear bands of gelatinolysis appeared on a blue background. Human MMP-2 and MMP-9 were used as a gelatin standard (Chemicon International Inc. Temecula, CA, USA). The images were scanned and quantification was performed using Image J programme. Full gels are shown in Supplementary Fig. 7.

### *In situ* zymography in ischaemic brain tissue

Mice were killed 3 h after reperfusion and perfused through the left ventricle with ice*-*cold PBS. Brains were removed, quickly frozen and kept at −80 °C. To assess gelatinase activity *in situ*, fresh sham and ischaemic brain slices (14 μm) were incubated with a reaction solution including DQ-gelatin conjugated with fluorescein (Life technologies, Carlsbad, CA) for 6 h at 37 °C. Sections were then washed three times with PBS and subsequently incubated with DAPI for nuclear staining. Images were captured with the Axio Imager A1 microscope, using AxioCam MRc camera and the AxioVision 4.8 programme (original magnification, × 40).

### Immunostaining for S1PR2 detection

Mice were anesthetized with Avertin and were perfused with cold PBS and subsequently with 4% PFA in PBS solution. The brains were removed, post-fixed with 4% PFA for 24 h and transferred to 30% sucrose solution. Frozen brains were cut with a thickness of 30 μm in a cryostat. Slices were preserved in 30%PEG 30% sucrose PBS. The brain slices were washed three times with Tris-buffered saline (TBS) and were then blocked with TBS-blocking solution (1% bovine serum albumin, 0.2% skim milk and 0.3% Triton X-100 in TBS) for 1 h and incubated with the following primary antibodies in TBS-blocking solution overnight on a shaker at 4 °C. Rabbit anti-S1P2R antibody (1:100, Proteintech Group Inc., Chicago, IL) and rat anti-CD31 antibody (1:100, BD Biosciences, Pharmingen, San Jose, CA) were used as primary antibodies. Sections were washed three times with TBS and then they were incubated with donkey anti-rabbit IgG Alexa-594 and goat anti-rat IgG Alexa-488 (1:250, Life Technologies, Molecular Probes, Grand Island, NY). Brain slices were stained with DAPI for 7 min and were mounted onto slides. Samples were observed on a Zeiss LSM 510 Meta Confocal microscope (Carl Zeiss Inc.). Images were captured using Zen LE software (Carl Zeiss). The specificity of the S1PR2 antibody was confirmed by lack of signal in the *S1pr2*^−/−^ brain sections.

### Brain endothelial cell culture and *in vitro* I/R studies

The mouse brain endothelial cell line bEnd.3 (passages 25–35; ATCC)^33^ was cultured in DMEM supplemented with 10% FBS. Passages 25–35 were used. hMVBEC (Cell Systems Corporation, Kirkland, WA) were cultured as recommended by the manufacturer. Passages 5–9 were used.

To mimic *in vitro* the metabolic and inflammatory stress of I/R injury, OGD and TNF-α activation studies were conducted.

Brain endothelial cells were exposed to hypoxia (1% O_2_)/aglycemia for 6 h in a hypoxia polymer glove box (Coy Lab Products Inc., MI). Then, the media was aspirated and replaced with complete growth media (which contains glucose) and cells were placed in a normoxic atmosphere. For the permeability studies, the media contained serum and changes in endothelial resistance after reperfusion were measured using the ECIS^34^. For the TNF-α activation studies, cells were stimulated with 5 ng ml^−1^ TNF-α in serum-free media containing growth factors (Cell Systems Corporation).

### Assessment of brain endothelial cell permeability

bEnd3.3 cells were grown to confluence in disposable electrode arrays containing gold film electrodes (Applied BioPhysics), previously coated with human fibronectin and collagen IV (50 μg ml^−1^) and exposed to hypoxia (1% O_2_)/aglycemia for 6 h in a hypoxia polymer glove box. Then, the media was aspirated and replaced with complete growth media (which contains glucose and 10% foetal bovine serum), cells were placed in a normoxic atmosphere and changes in endothelial resistance on reperfusion were measured using the ECIS^34^

### MMP activity in brain endothelial cell supernatants

For *in vitro* detection of MMP-2 and MMP-9 activity, conditioned media from hBMVEC subjected to TNF-α treatment were used. hBMVEC were grown on 12-well plates. Cells were treated with TNF-α (5 ng ml^−1^) in serum-free complete growth media (Cell Systems Corporation) for 24 h. Conditioned media were collected, spun down, 10 μl of supernatants were mixed with equal amounts of SDS sample buffer and gelatin zymography was conducted as described above.

### Primary cortical neuron isolation and *in vitro* I/R studies

Mouse (C57BL6) E16.5 embryos were used for primary cortical neuron isolation as previously described^68^. Briefly, the cortices were collected and incubated with 0.25% trypsin for 15 min at 37 °C for tissue digestion. Foetal bovine serum was added to stop the trypsin activity. After centrifugation, the supernatant was discarded and DMEM complete media containing 10% FBS and antibiotics were added to the cells. The cell suspension was passed through 70 μm cell strainer (BD Biosciences, Falcon) and was plated on poly-L-lysine (Sigma Aldrich, St Louis, MO) coated dishes with DMEM complete media. The next day, the medium was changed to Neurobasal media containing B27 supplement, antibiotics and GlutaMAX (Life Technologies). At 7 days *in vitro*, the cells (>95% NeuN positive) were used for the *in vitro* I/R injury assays. Neurons were subjected to hypoxia (1% O_2_)/aglycemia for 4 h in a hypoxia polymer glove box. Then, the media was aspirated and replaced with complete growth media (which contains glucose) in the presence or absence of vehicle, α-PBN or JTE013 and cells were placed in a normoxic atmosphere. Cells were harvested for RNA isolation, quantitative reverse transcription–PCR analysis (qRT–PCR), 6 or 24 h after *in vitro* reperfusion to determine S1PR2 mRNA levels. LDH activity was assessed 24 h after reperfusion. To mimic the oxidative stress characteristic of I/R injury, neurons were treated with 30 μM H_2_O_2_ in the presence or absence of vehicle, α-PBN or JTE013 for 24 h and LDH activity was assessed in neuronal supernatants.

### LDH assay

Neuronal cytotoxicity was assessed by measuring LDH activity in the media, 24 h after *in vitro* I/R injury or H_2_O_2_ treatment, using the Cytotoxicity Detection Kit (Roche Applied Science), as instructed by the manufacturer. Data were plotted as fold change compared with non-treated control cells. α-PBN was used as a positive control for oxidative stress-induced cell death blocker^35^.

### Mixed glial cell culture and activation

Mixed glial cell culture was prepared using the mouse brains from postnatal day 2 mouse. Briefly, cerebral cortices were dissected, trypsinized with of 0.25% trypsin–EDTA in Hank's Balance Salt Solution (HBSS) and were incubated with trypsin (Thermo scientific) and DNase (Worthington) for 15 min at 37 °C. Foetal bovine serum was added to the cell suspension to stop trypsin digestion. Cell suspension was centrifuged and the pellet was resuspended with DMEM containing 20% FBS and antibiotics. Cell suspension was filtered with a 100-μm cell strainer (BD Falcon) into another 50 ml conical tube. Cells were plated onto six-well plates, which were pre-coated with poly-D-lysine. Three days after plating, the media was changed to DMEM containing 10% FBS and antibiotics. Cells were maintained in DMEM containing 10% FBS and antibiotics at 37 °C with 5% CO2, with a medium change every 3 days. At day 10 after plating, mixed glial cells were treated with 5 ng ml^−1^ TNF-α to mimic the pro-inflammatory environment of I/R injury. About 24 h later, MMP activity was assessed in the supernatants as described below and glial cells were harvested for RNA isolation and qRT–PCR analysis to determine S1PR2 mRNA levels.

### S1PR2 Immunohistochemistry

Immunohistochemistry for S1PR2 was performed on an automated stainer (Leica Bond III, Leica Biosystems, Buffalo Grove, IL) using an anti-human S1PR2 rabbit polyclonal antibody (catalogue # HPA014307, Sigma Aldrich) at a final dilution of 2 μg ml^−1^. Cell pellets and 5-μm formalin-fixed paraffin-embedded tissue sections were deparaffinized and processed using heat-induced epitope retrieval with an EDTA-based buffer (Leica # AR9640) for 20 min and incubated with primary antibody for 30 min at room temperature. Post-primary reagent (polymer anti-rabbit poly-horseradish peroxidase-IgG, <25 μg ml^−1^, Leica # DS9800) was incubated for 10 min at room temperature and staining was visualized with diaminobenzidine for 10 min (Leica # DS9800). Pictures were taken with a × 60 objective using Olympus BX41 Camera and CellSens Entry Imaging Software. Cases were retrieved from Brigham and Women's Department of Pathology archives and this work was approved by the Institutional Review Board (Protocol #2013P001431).

### RNA isolation

To prepare mouse organ tissue lysates, mice were perfused with PBS containing 2 mM EDTA, organs were removed and snap frozen in liquid nitrogen. Organs were homogenized and total RNA was prepared using RNeasy Mini Kit (Qiagen, Valencia, CA) with RNase-free DNase treatment (Qiagen) to remove the genomic DNA.

### TaqMan qRT–PCR analysis

To generate complementary DNA (cDNA), 100 ng of RNA was reverse transcribed using random primers and SuperScript II RT-polymerase (Invitrogen, Carlsbad, CA). Primers were designed using the Primer Express oligo design programme software (Applied Biosystems, Foster City, CA). All primer sets were subjected to rigorous database searches to identify potential conflicting transcript matches to pseudogenes or homologous domains within related genes. PCR primer sequences for target molecules were previously described^21^ and shown in Supplementary Table 2. Amplicons generated from the primer set were analysed for melting point temperatures using the first derivative primer melting curve software supplied by Applied BioSystems. Real-time quantitative PCR was performed using the SYBR Green I assay on the ABI 7500 Sequence Detection System (Applied Biosystems). PCR reactions for each cDNA sample were performed in duplicate and copy numbers were calculated using standard curves generated from a master template as previously described^69^. Briefly, each cDNA template was purified, precisely quantified and 10-fold serially diluted to produce a series in which the lowest-concentration dilution has a single copy per microlitre. To generate a standard curve, the threshold cycle (Ct) values for each dilution in the template series are plotted as a function of the logarithm of the known input template numbers and a linear trendline is fitted to the data.

### Statistical analyses, randomization and blinding

All values reported are mean±s.e.m. *P* values were calculated with GraphPad Prism software, using (i) one-way ANOVA followed by Newman–Keuls test or (ii) Student’s *t*-test when comparing two groups. The criterion for statistical significance was set at *P*<0.05.

All animal experiments used randomization to treatment groups and blinded assessment^70^.

## Additional information

**How to cite this article:** Kim, G. S. *et al.* Critical Role of sphingosine-1-phosphate receptor-2 in the Disruption of Cerebrovascular Integrity in Experimental Stroke. *Nat. Commun.* 6:7893 doi: 10.1038/ncomms8893 (2015).

## Supplementary Material

Supplementary Information Supplementary Figures 1-7, Supplementary Tables 1-2, Supplementary Methods and Supplementary References

## Figures and Tables

**Table 1:** Physiological variables in *S1pr2* ^*+/+*^ and *S1pr2* ^*−/−*^ mice.

**Time points**	**Parameters**	***S1pr2*** ^***+/+***^	***S1pr2*** ^***−/−***^
10 min before MCAO	Arterial O_2_ saturation (S_p_O_2_, %)	97.0±0.6	98.4±0.2
	Heart rate (b.p.m.)	493.0±9.2	520.9±31
	Pulse distention (μm)	13.4±4.5	18.3±8.3
	Respiratory rate (br.p.m.)	110.1±11.1	109.1±5.1
10 min after MCAO	Arterial O_2_ saturation (S_p_O_2_, %	97.0±0.6	98.2±0.3
	Heart rate (b.p.m.)	531.3±13.8	524±13.5
	Pulse distention (μm)	19.3±5.8	15.7±3.7
	Respiratory Rate (br.p.m.)	104.5±3.5	115.7±8.3
10 min after reperfusion	Arterial O_2_ saturation (S_p_O_2_, %)	96.8±1.1	98.1±0.2
	Heart rate (b.p.m.)	496.6±14.76	525.6±11
	Pulse distention (μm)	16.6±1	13.0±3.3
	Respiratory rate (br.p.m.)	107.8±5.8	112.2±5.2

bpm, beats per minute; br.p.m., breaths per minute; MCAO, middle cerebral artery occlusion.

Pulse distention is a surrogate for pulse pressure (pulse pressure=systolic pressure−diastolic pressure). JTE013 (30 mg kg^−1^) or vehicle was administered after reperfusion. No significant differences were observed in the physiological parameters (arterial O_2_ saturation, heart rate, pulse distention and respiratory rate) between wild-type and *S1pr2*^*−/−*^ mice, before, during or after MCAO.
