# Supplementary material for: Critical role of sphingosine-1-phosphate receptor-2 in the disruption of cerebrovascular integrity in experimental stroke
Source: Nat Commun. 2015 Aug 5;6:7893. doi: 10.1038/ncomms8893 (PMC4587559; doi:10.1038/ncomms8893)
Supplement: Supplementary Information — Supplementary Figures 1-7, Supplementary Tables 1-2, Supplementary Methods and Supplementary References [file ncomms8893-s1.pdf]

SUPPLEMENTARY INFORMATION

Supplementary Figure 1

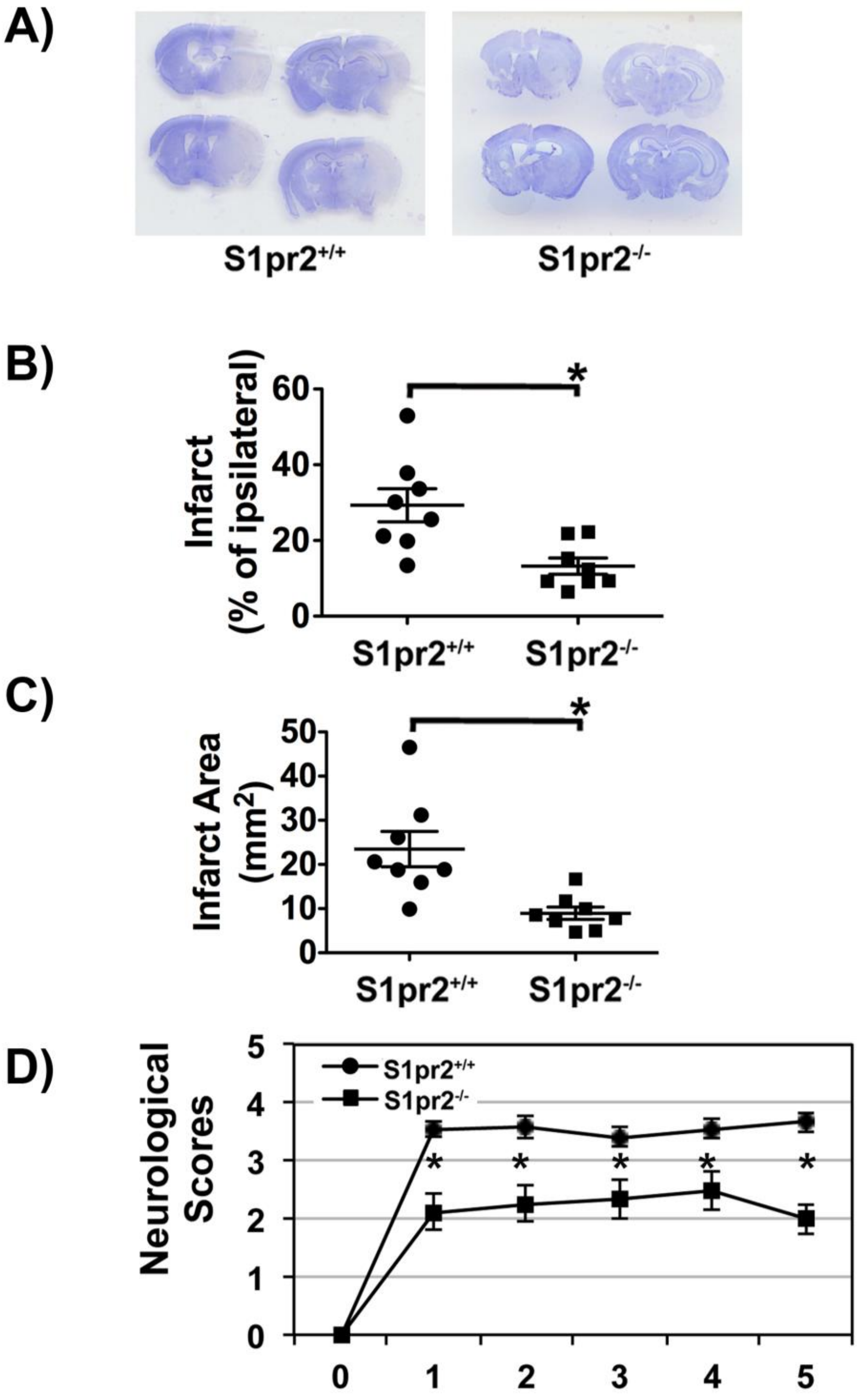

Supplementary Figure 1. Long-term protection studies. 45 minutes of ischemia was induced in wild type (*S1pr2*$^{+/+}$) and *S1pr2*$^{-/-}$ by MCAO. A) 5 days later brains were harvested and cresyl violet staining was conducted. Representative pictures are shown. B) and C) Infarct areas from 4 brain coronal sections were quantified by image analysis (C) and were plotted as percentage of the ipsilateral hemisphere (B). The individual values and the mean ± SEM are shown. N=8 from 8 independent experiments. *$p<0.05$. D) Neurological scores were assessed 1, 2, 3 4, and 5 days after reperfusion in wild type (*S1pr2*$^{+/+}$) and *S1pr2*$^{-/-}$ mice. Values are mean±SEM. *$p<0.05$.

Supplementary Figure 2

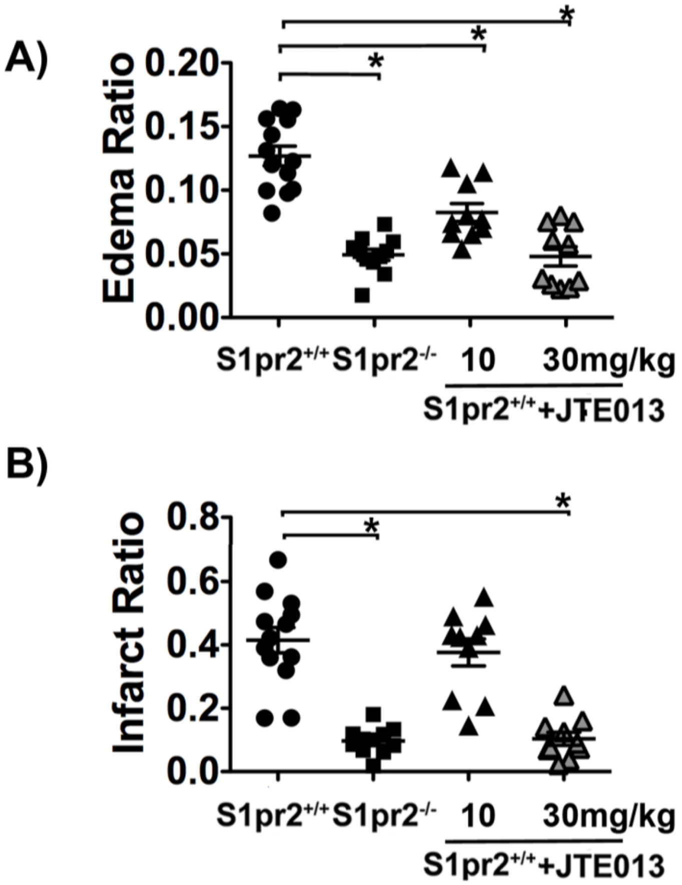

Supplementary Figure 2. Effective dose of the S1PR2 antagonist, JTE013, in experimental stroke. Ischemia (90 min.) was induced in wild type (*S1pr2*$^{+/+}$) and *S1pr2*$^{-/-}$ by MCAO. 10 minutes after reperfusion mice received vehicle or the S1PR2 antagonist, JTE013 (10 mg/kg or 30mg/kg), by gavage. 24 hours later, brains were harvested and TTC staining was conducted. A) Edema and B) Infarct ratios were calculated by image analysis and reported as a ratio of the non-ischemic hemisphere. Infarct ratios were corrected for edema. The individual values and the mean ± SEM are shown. N=10-13 from 8 independent experiments. *p<0.05.

Supplementary Figure 3

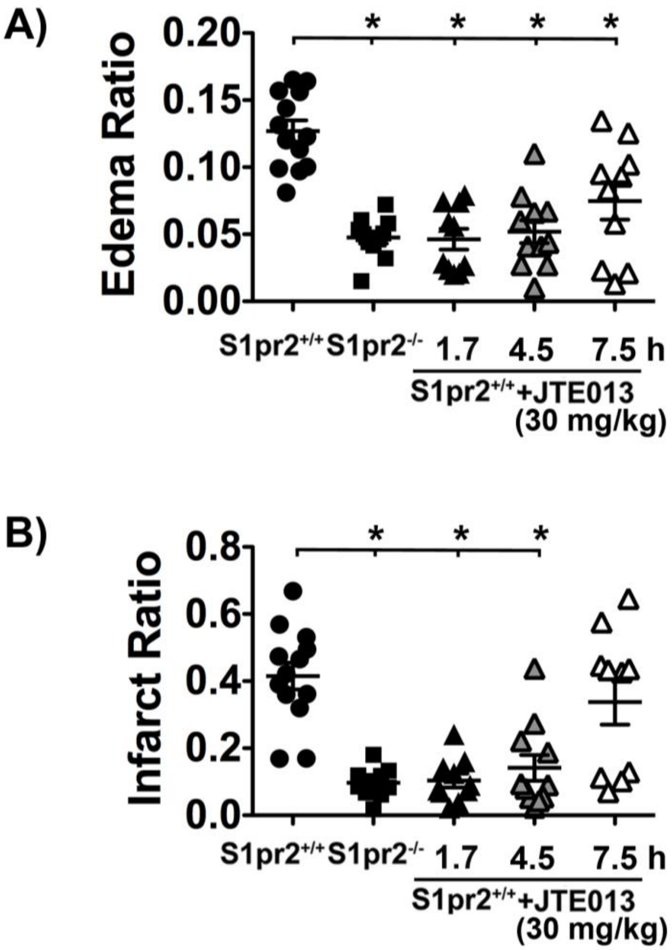

Supplementary Figure 3.Therapeutic time window of effectiveness for JTE013. Transient focal ischemia (90 min.) was induced in wild type (*S1pr2*$^{+/+}$) and *S1pr2*$^{-/-}$ by MCAO. 1.7, 4.5 or 7.5 hours after the onset of ischemia mice received vehicle or the S1PR2 antagonist, JTE013 (30mg/kg), by gavage. A) Edema and B) Infarct ratios were calculated by image analysis and reported as a ratio of the non-ischemic hemisphere. Infarct ratios were corrected for edema. The individual values and the mean ± SEM are shown. N=10-13 from 8 independent experiments. *$p < 0.05$.

Supplementary Figure 4

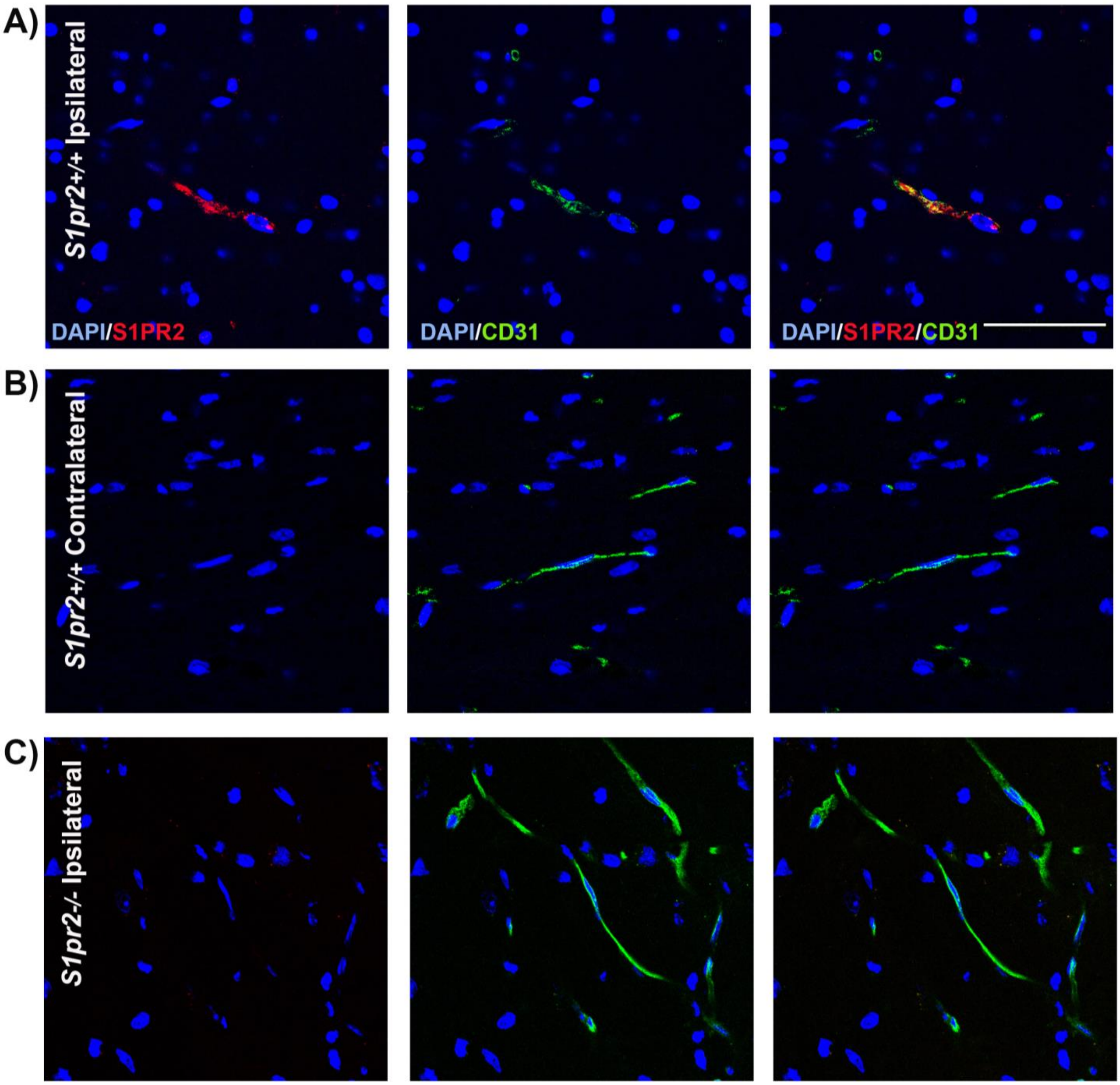

Supplementary Figure 4. S1PR2 immunofluorescence in ischemic *striatum*. 6 hours after tMCAO, brains were harvested and S1PR2 (red channel) and CD31 (green channel) immunofluorescence analysis was conducted in brain sections from the ipsilateral (A) or contralateral (B) hemisphere of wild type mice or ipsilateral hemisphere of *S1pr2*$^{-/-}$ mice (C). Representative pictures of the damaged *striatum* at the level of bregma +1.2mm to +0.8mm, rostrally, as indicated in Figure 1G, are shown (n=5-6 per group). S1PR2 was detected in cerebral microvessels only in the damaged area (ipsilateral hemisphere, A) but not in the contralateral hemisphere (B) or the ipsilateral hemisphere of *S1pr2*$^{-/-}$ mice (C). Scale bar 50 μm.

Supplementary Figure 5

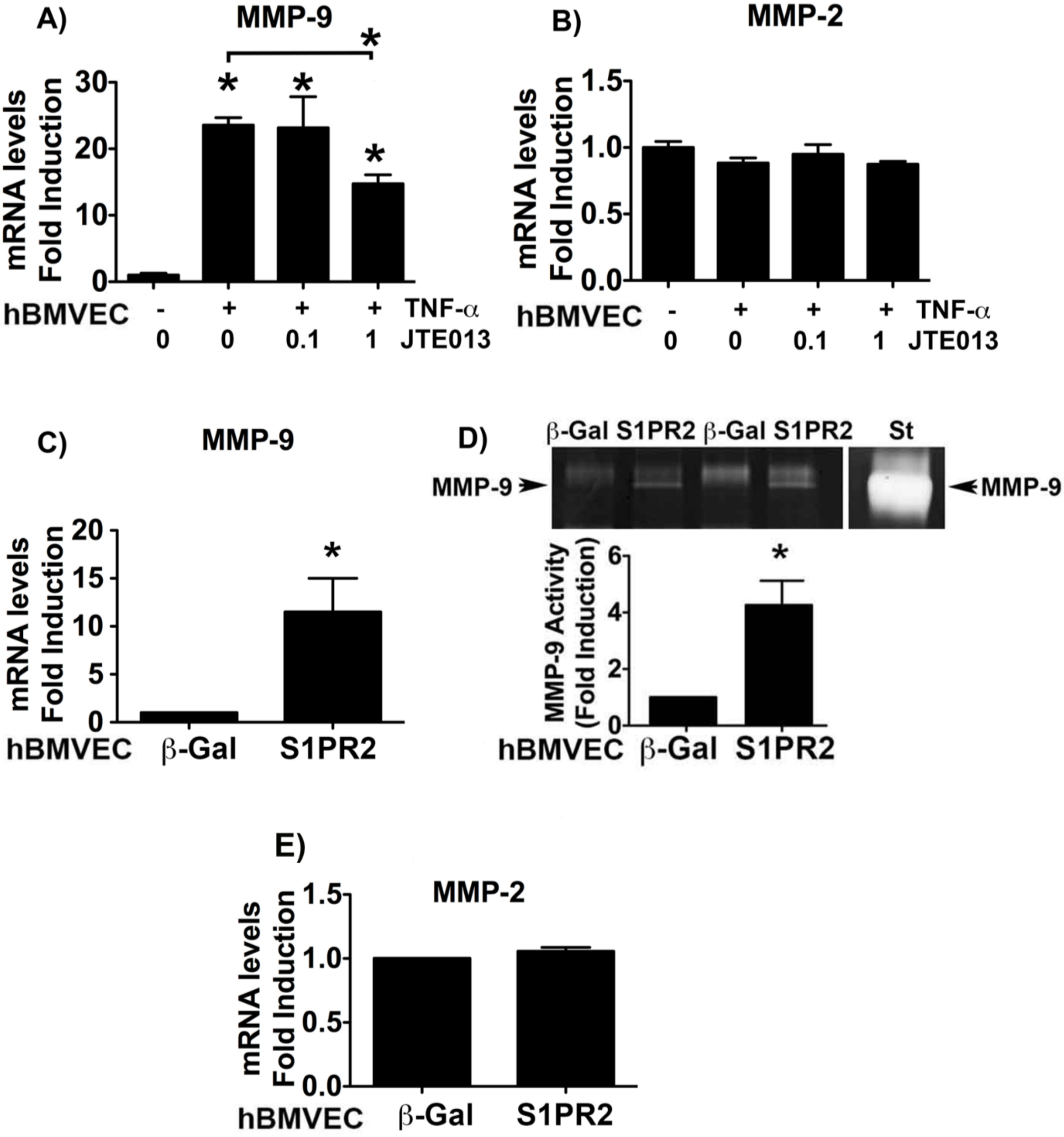

Supplementary Figure 5. Regulation of MMP-9 and MMP-2 mRNA levels and activity by S1PR2 in endothelial cells. A, B) hBMVEC were stimulated with 5ng/mL TNF-α for 6 hours in the presence of absence of JTE013 at the indicated concentrations (μM). C-E) hBVMEC were transduced with β-Galactosidase (β-Gal) or S1PR2 adenoviruses (20 M.O.I) for 24 hours. A-C, E) RNA was isolated, MMP-9 and MMP-2 mRNA levels were determined by reversed transcription and quantitative PCR analysis and normalized by 18S RNA. Fold induction *vs* non-stimulated cells is shown. Values are mean±SEM, n=3-4. *P<0.05 TNF-α *vs* vehicle or S1PR2 *vs* β-Gal, and when indicated TNF-α+JTE013 *vs* TNF-α+Vehicle. D) Upregulation of S1PR2 by adenoviral transduction increases MMP-9 activity in the conditioned media. A representative zymography with two independent experiments is shown. Values are mean ± SEM. n=4-5 from 4 independent experiments. * P≤0.05 S1PR2 vs β-Gal.

Supplementary Figure 6

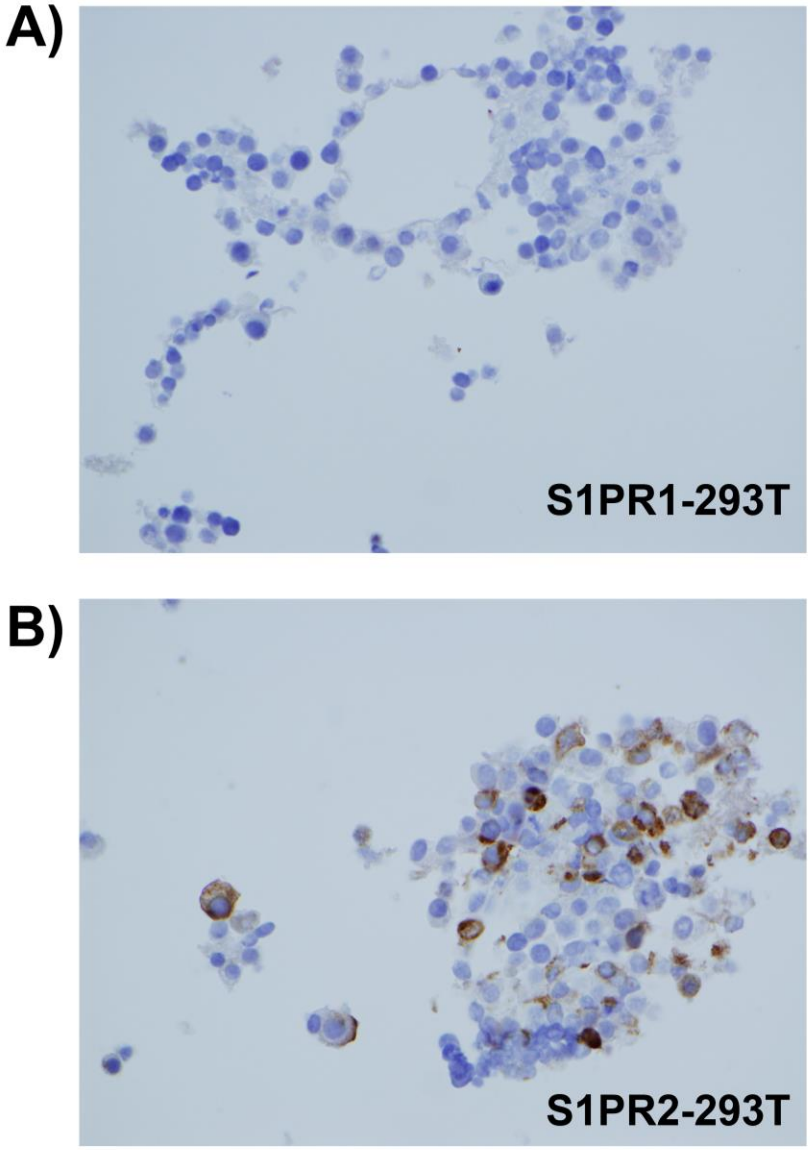

Supplementary Figure 6. **Validation of the anti-S1PR2 antibody for immunohistochemistry**. Representative images of S1PR2 immunohistochemistry of transiently transfected HEK-293T cells. HEK-293T cells were transiently transfected with either a pcDNA3.1-S1PR1 (A) or pcDNA3.1-S1PR2 (B). Immunohistochemistry with the anti-S1PR2 antibody was performed on cell pellets as described in the methods section using the same staining protocol as for the clinical cases. Note the S1PR2 positivity in S1PR2-transfected cells but not the in cells transfected with S1PR1. Vector control (pcDNA3.1) transfected cells were also negative (not shown).

Supplementary Figure 7

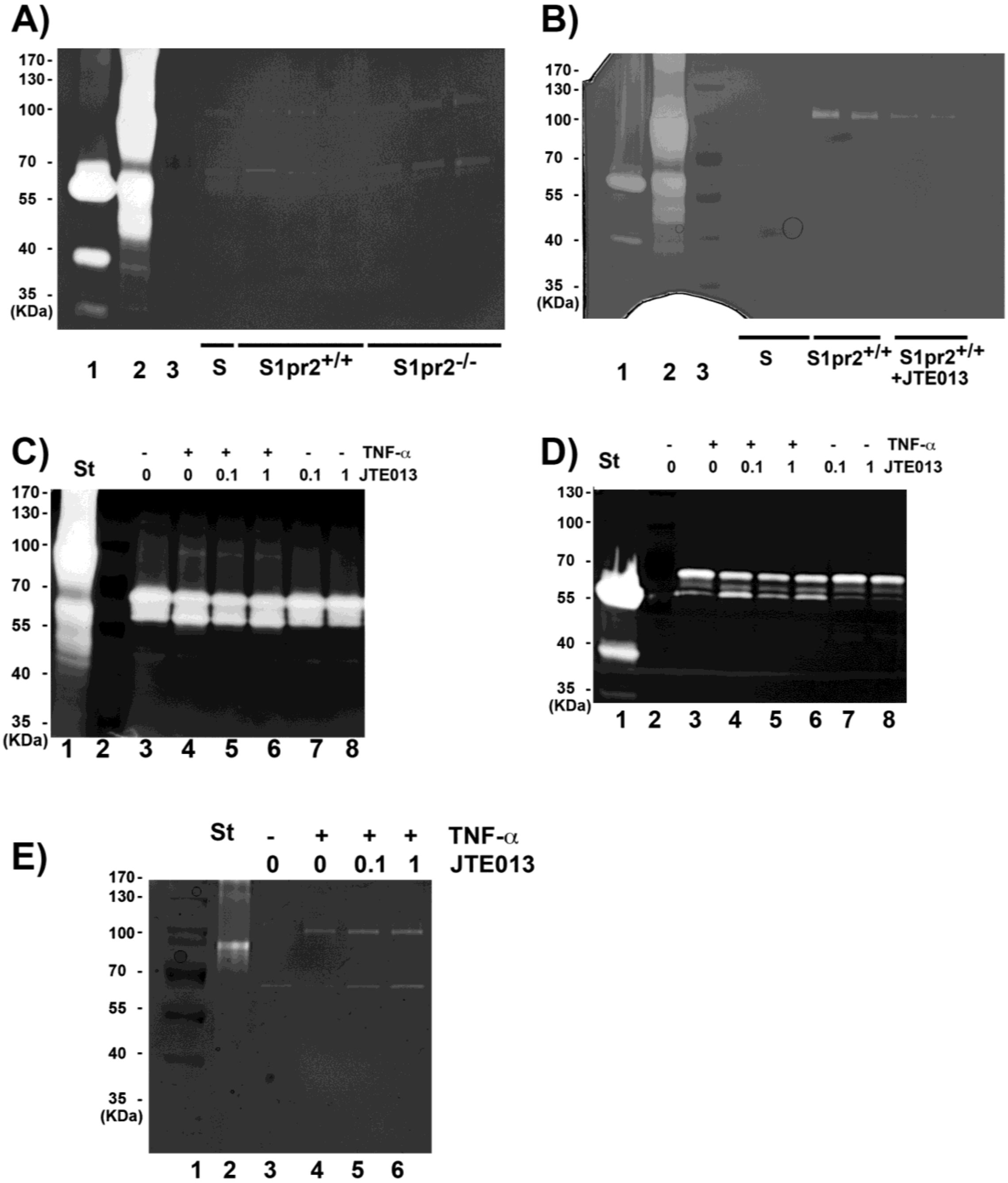

Supplementary Figure 7. Uncropped scanned images of gelatin zymographies. A) Figure 4A, left panel. Lane 1: MMP-2 standard, lane 2: MMP-9 standard, lane 3: molecular weight marker. B) Figure 4A, right panel. Lane 1: MMP-2 standard, lane 2: MMP-9 standard, lane 3: molecular weight marker. C) Figure 6E, top panel (MMP-9, 48-hour development). Lane 1: MMP-9 standard, lane 2: molecular weight marker. D) Figure 6E, bottom panel (MMP-2, same samples as in C, 24-hour development) Lane 1: MMP-2 standard, lane 2: molecular weight marker. E) Figure 7F. Lane 1: molecular weight marker, lane 2: MMP-9 standard.

SUPPLEMENTARY TABLE 1. Available clinical features of autopsy cases

Case no.	Clinical characteristics
1	40 year-old man with history of idiopathic end stage emphysema s/p 2 lung transplants. Patient died from hypercarbic respiratory failure.
2	21 year-old woman with sickle cell disease and a history of recurrent vaso-occlusive episodes (pain crises). Patient died 18 hours after presumed arrhythmia in setting of pain crisis, acute renal failure, ischemic hepatitis and profound metabolic acidosis.
3	33 year-old man with history of idiopathic dilated cardiomyopathy diagnosed 5 years prior to death. Patient died due to progressive heart failure, respiratory failure and acute renal failure.
4	58 year-old man with dyslipidemia and previous history of substance abuse. Unknown cause of death.
5	69 year-old woman with insulin dependent diabetes mellitus, hypertension, end stage renal disease, atherosclerosis, and hypercholesterolemia. The patient had bilateral anterior cerebral artery-middle cerebral artery and middle cerebral artery-posterior cerebral artery watershed infarcts. Patient died due to complications from stroke.

SUPPLEMENTARY TABLE 2. Primer sequences for quantitative PCR analysis

**Target Genes**	**Sequence of Primers**
Mouse S1PR2	F: ATGGGCGGCTTATACTCAGAG<br>R: GCGCAGCACAAGATGATGAT
Human S1PR2	F: TGGCCGCCTCCGATCT<br>R: GAGAGCAAGGTATTGGCTACGAA
Human MMP-9	F: CCTGGAGACCTGAGAACCAATC<br>R: GATTTCGACTCTCCACGCATCT
Human MMP-2	F: TTGATGGCATCGCTCAGATC<br>R: CTTGTCACGTGGCGTCACA
Mouse IL-1β	F: GCCCATCCTCTGTGACTCATG<br>R: GGAGCCTGTAGTGCAGCTGTCT

SUPPLEMENTARY METHODS

Adenoviral transduction

For gain-of-function studies, hBMVEC were transduced with β-galactosidase or S1PR2 adenovirus (at 20 multiplicity of infection, M.O.I.). At this low M.O.I the levels of S1PR2 mRNA were similar to S1PR1 mRNA levels, as we have previously described [1,2]. 20 hours after adenoviral transduction, medium was replaced with serum-free complete growth media and 24 hours later, gelatinase activity was determined in the supernatants.

Cresyl Violet Staining

After the neurological score evaluation at 5 days after tMCAO (45 minutes of ischemia), mice were anesthetized with Avertin and were perfused with cold PBS and subsequently with 4% PFA in PBS solution. The brains were removed, postfixed with 4% PFA for 24 h and transferred to 30% sucrose solution. Frozen brains were cut with a thickness of 30 µm in a cryostat. Cresyl violet staining was performed to evaluate the neuronal injury at 5 days after tMCAO.

SUPPLEMENTARY REFERENCES

1. Sanchez, T. *et al.* Induction of vascular permeability by the sphingosine-1-phosphate receptor-2 (S1P2R) and its downstream effectors ROCK and PTEN. *Arterioscler Thromb Vasc Biol* **27**, 1312-1318 (2007).
2. Zhang, G. *et al.* Critical role of sphingosine-1-phosphate receptor 2 (S1PR2) in acute vascular inflammation. *Blood* **122**, 443-455, doi:10.1182/blood-2012-11-467191 (2013).
